# Substantial effect of phytochemical constituents against the pandemic disease influenza—a review

**DOI:** 10.1186/s43094-021-00269-5

**Published:** 2021-06-12

**Authors:** A. Brindha Devi, R. Sarala

**Affiliations:** Department of Botany, Periyar EVR College (Autonomous), (Affiliated to Bharathidasan University, Trichy-24), Trichy-620 023, Tamil Nadu, India

**Keywords:** Influenza, Medicinal herb, Phytochemical, Primary metabolite, Secondary metabolite, Drug

## Abstract

**Background:**

Influenza is an acute respiratory tract infection caused by the influenza virus. Vaccination and antiviral drugs are the two methods opted to control the disease. Besides their efficiency, they also cause adverse side effects. Hence, scientists turned their attention to powerful herbal medicines. This review put focus on various proven, scientifically validated anti-influenza compounds produced by the plants suggested for the production of newer drugs for the better treatment of influenza and its related antiviral diseases too.

**Main body:**

In this review, fifty medicinal herb phytochemical constituents and their anti-influenza activities have been documented. Specifically, this review brings out the accurate and substantiates mechanisms of action of these constituents. This study categorizes the phytochemical constituents into primary and secondary metabolites which provide a source for synthesizing and developing new drugs.

**Conclusion:**

This article provides a summary of the actions of the herbal constituents. Since the mechanisms of action of the components are elucidated, the pandemic situation arising due to influenza and similar antiviral diseases can be handled promisingly with greater efficiency. However, clinical trials are in great demand. The formulation of usage may be a single drug compound or multi-herbal combination. These, in turn, open up a new arena for the pharmaceutical industries to develop innovative drugs.

## Background

Influenza virus infections remain a major formidable disease of man defying control. It is a widespread disease of man occurring in epidemics and pandemic forms [1]. Influenza severity varies from mild to extreme and is determined by the virus type and its host [2].

Influenza viruses belong to the Orthomyxoviridae family which comprises seven genera Alpha influenza virus, Beta influenza virus, Delta influenza virus, Gamma influenza virus, Isa virus, Quaranja virus, and Thogotovirus [3, 4]. Influenza A, B, D, and C viruses belong to Alpha, Beta, Delta, and Gamma genera respectively.

Influenza A virus (IAV) hosted a wide range of species including wild aquatic birds, humans, poultry, pigs, horses, and sea mammals [5] which cause epidemics and pandemic conditions [6]. Influenza B virus (IBV) infects humans and seals [5, 7]. Influenza C virus (ICV) infections have been documented in humans and swine [5, 7]. Influenza B and C viruses create epidemics but lack pandemic potential [8]. Influenza D virus (IDV) infection was observed in farmed cattle, goats, pigs, and buffalo [9]. Serological evidence detects the presence of the virus in humans [10] but the investigation of transmission remains unclear [7, 11].

The World Health Organization (WHO) along with the National Influenza Centers (NIC) and Centers for Disease Control and Prevention (CDC) strengthen global influenza surveillance during every seasonal outbreak [12, 13]. These authorized centers monitor global influenza circulation, detecting the emergence of new strains followed by the recommendation of vaccine usage [12, 13]. Many ongoing research activities like vaccine preparation, clinical trial of new drugs, and Traditional and Complementary medicine (T&CM) using herbs are carried worldwide to anticipate their work. Traditional and Complementary medicine (T&CM) role is indispensable for the prevention and management of chronic diseases [14] and WHO estimates 65–80% of the world population rely on it [15]. Interestingly, 34 countries include herbal medicines in their national essential medicines list (NEML) [14]. Hence, herbal medicines are always in great demand for the ailment of severe diseases.

This review article enlists several anti-influenza potential herbs along with their phytochemical constituents showing multi-targeted action against influenza. This study significantly provides experimentally validated phytoconstituents showing the evidence of efficacy against influenza.

## Main text

### Influenza viruses—an overview

Influenza viruses are spherical (100 nm in diameter) or filamentous (300 nm in length) in shape [16]. They have a central core surrounded by an envelope. The central core region has RNA segments covered with a nucleocapsid protein (NP). The entire core is surrounded by a matrix protein (M1) consequently covered by a lipid bilayer from which two spiked surface glycoproteins hemagglutinin (HA) and neuraminidase (NA) arise. The glycoprotein HA is a tetramer whereas NA is a trimeric molecule [16].

IAV contains 8 RNA segments coding 7 structural proteins (PB1, PB2, PA, HA, NA, NP, and M1) and 3 nonstructural proteins (NS1, NS2, and M2) [17]. The structural proteins HA and NA are the antigenic determinants [18] and the main targets for antiviral drugs [17]. Based on these proteins, influenza A is classified into different subtypes hemagglutinin (H1–H18) and neuraminidase (N1–N11).

IBV contains 8 RNA genome segments where the nonstructural protein M2 is replaced by BM2. Influenza B has 2 lineages B/Yamagata and B/Victoria and they are not classified into subtypes [19].

ICV has 7 genome segments with a single trimeric glycoprotein called hemagglutinin esterase fusion [HEF] which equalize the function of HA and NA protein [20] along with a minor envelope protein CM2 [21]. Currently, the influenza C virus has 6 lineages [22].

**Fig. 1:**
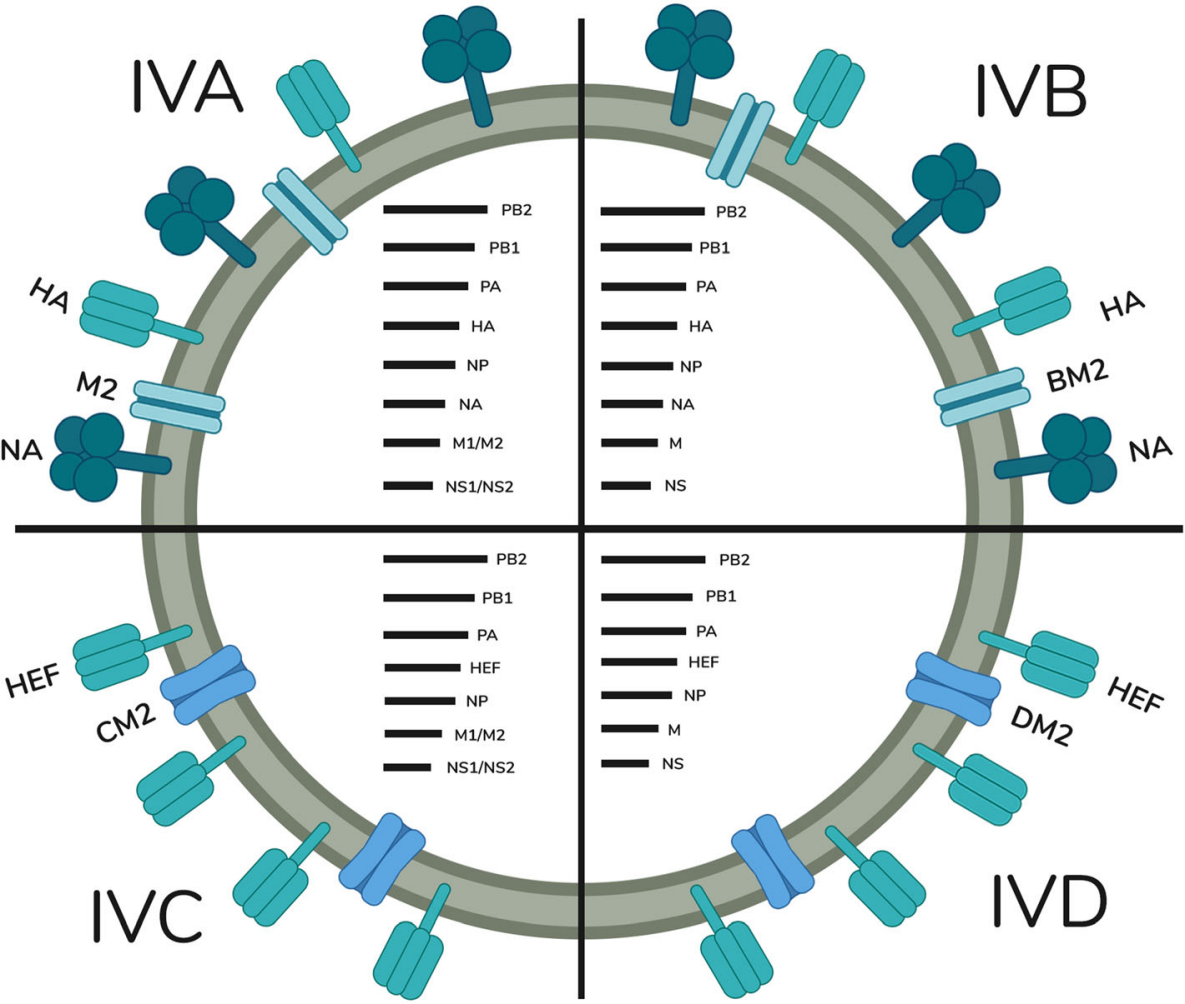
Structure of influenza viruses A, B, C, and D with their genomic arrangements

IDV is a newly emerging virus with 7 genome segments with a matrix protein DM1 and ion channel protein DM2 [23] in association with glycoprotein hemagglutinin esterase fusion (HEF) like influenza C virus. At present, it has 2 distinct co-circulating lineages [24]. Figure 1 displays the structural description of influenza viruses.

### Antigenic variation

Influenza viruses profoundly alter their surface glycoprotein resulting in an antigenic variation [25, 26] which exhibits in two forms antigenic drift and antigenic shift [26]. Antigenic drift is a small, gradual change of the surface antigen due to genomic point mutations [27] forming new strains of the virus, cause epidemics [28]. Random occurrence of antigenic drift is observed in influenza A, B [24], and C viruses [29]. In contrast, drastic antigenic change has not been recorded for the IDV [23]. Hence, its epidemic nature also remains undetermined. Antigenic shift occurs when a sudden extreme and profound change in genome reassortment results in the formation of a new subtype [28]. It is observed only in influenza A virus which leads to the pandemic condition [30].

### Vaccination remains challenge

Vaccines are substances that provide immunity and protection against a particular infectious disease. Introducing vaccines into the body refers to vaccination and getting immunity through vaccination is immunization [31]. Every year, 3 million deaths are prevented by vaccines, and through vaccinations, the human life span has increased [32]. This shows the promising notice that vaccinations are the best way of preventing the serious effect of the dangerous disease.

Influenza is often called flu and flu vaccination is a principal tool for the prevention of influenza severity. Influenza A viruses cause pandemics whereas influenza A and B viruses cause seasonal epidemics. The World Health Organization [WHO] assisted with the National Influenza Centers [NIC] and Centers for Disease Control and Prevention (CDC) makes the recommendation for two different vaccine formulations every year, one for the Northern and one for the Southern Hemisphere [33].

**Fig. 2:**
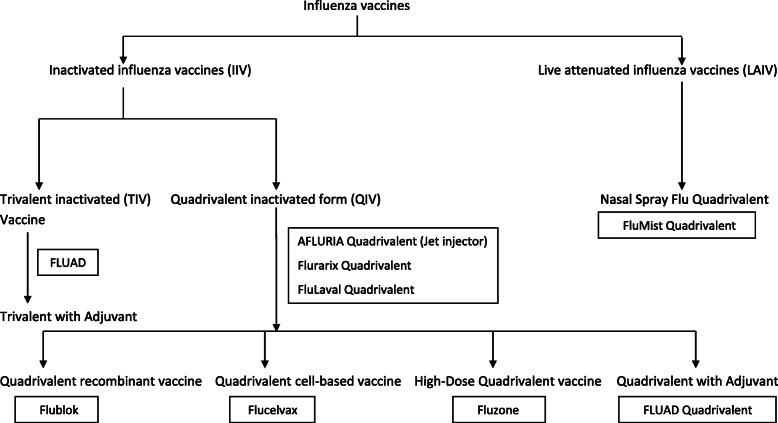
Influenza vaccine types and drug brand names recommended by CDC

In general, influenza vaccines are prepared in two forms as inactivated influenza vaccines (IIV) and live attenuated influenza vaccines (LAIV) [34]. Intramuscular injection of the flu vaccine is referred to as a flu shot which may be in trivalent inactivated (TIV) or quadrivalent inactivated form (QIV). The TIV is comprised of two influenza A strains and one influenza B lineage while QIV includes two influenza A strains and two influenza B lineages [35]. Figure 2 displays influenza vaccine types and drug brand names recommended by the CDC.

TIV is a standard vaccine recorded as safe and creates immunogenicity among children of 6 through 35 months of age and elderly of 65 and above [36, 37]. QIV under the brand names of AFLURIA Quadrivalent, Fluarix Quadrivalent, and FluLaval Quadrivalent consists of embryonated egg-grown medium vaccines, recommended for children of 6 months to 3 years, and older. People with egg allergy adapt egg-free quadrivalent vaccines like quadrivalent recombinant vaccine and quadrivalent cell-based vaccine. Quadrivalent recombinant vaccines are produced by recombinant technology where the vaccines obtained from mammalian cell culture produce quadrivalent cell–based vaccine [38]. Recently, the U.S. Food and Drug Administration (FDA) approved Flublok Quadrivalent recombinant vaccine and Flucelvax Quadrivalent cell–based vaccine for use in adults 18 years and older [39].

The High-Dose Quadrivalent vaccine with the brand name Fluzone contains four times the antigen intended to give protection for elderly people [40]. A recent study reveals that the high-dose vaccine increases the immune response [41]. AFLURIA Quadrivalent® is the only vaccine approved for use with a jet injector that comes in multi-dose vials recommend for 18- to 64-year-old people [42].

Adjuvants are the compounds that enhance immunity. Currently, adjuvants like Alum, MF59 (oil-in-water emulsion), AS03 (oil-in-water adjuvant), AF03 (oil-in-water adjuvant containing squalene, montane 80, and eumulgin b1 ph), virosomes (phospholipids), and heat-labile enterotoxin (LT) [43] are used in TIV and QIV as FLUAD and FLUAD Quadrivalent.

The Nasal Spray Flu Vaccine under the brand name of FluMist Quadrivalent is a live attenuated influenza vaccine [LAIV] is recommended by FDA in 2012 for people ages 2 to 49. Even though the nasal vaccine stimulates the long-standing immune response, CDC not recommends it during the 2016–2017 and 2017–2018 flu seasons. This is due to its less effectiveness than IIV during the 2009 pandemic. After obtaining positive protection data of LAIV, CDC recommends it for the 2018–2019 season [44].

Mutations make the influenza viruses change over time. Hence, the genetic materials also change and new subtypes are evolved [45] which often replace the older strain [46]. It is a fact that influenza vaccines save countless lives and prevent pandemics but annual reformulation in vaccine preparation for the new emerging strains remains a challenge for the scientist [47].

Vaccine preparation takes a long time since it relies on the official declaration of the prevailing viral strain by WHO [48]. Marketing vaccine strains do not give enough protection against novel emerging strains [49]. At present, vaccines are prepared by three production technologies: egg-based, cell culture, and recombinant methods. The majority of the vaccine production is egg-based which is time-consuming and mismatching from the available and prevailing strains [48]. To overcome this, cell culture and recombinant methods of vaccine production are recently adapted, which are still in need of appropriate infrastructure to attain the goal.

Global demand for vaccines and shortage of production are growing exponentially. This is noted from the recent survey indicating that the requirement of seasonal vaccine is 1.504 billion doses but the production capacity is decreased to 1.467 billion doses [46, 50]. The selectivity of vaccines for elderly people is also biased since vaccine efficacy differs by age and chronic diseases [51]. Hence, these conditions make developing universal influenza vaccines a big task.

### Conventional drugs for the disease

Antiviral drugs are another alternative way to control and manage seasonal and post-exposure influenza [52]. They are classified into three broad categories as M2 inhibitors, neuraminidase inhibitors, and nucleoprotein inhibitors [53]. The antiviral drugs Amantadine and Rimantadine fall under M2 inhibitors, Rapivab (Peramivir), Relenza (Zanamivir), and Tamiflu (Oseltamivir Phosphate) are the neuraminidase inhibitor drugs whereas Xovluza (baloxavir marboxil) is a Nucleoprotein-inhibitor drug.

The M2 ion channel–inhibitor drugs (Amantadine and Rimantadine) are specifically used to treat influenza A infection. Currently, CDC has not recommended these drugs since they create resistance against many strains [52].

In the present scenario, CDC recommends four FDA (Food and Drug Administration)-approved anti-influenza drugs, namely Rapivab (Peramivir), Relenza (Zanamivir), Tamiflu (Oseltamivir Phosphate), and Xovluza (baloxavir marboxil) [54]. These drugs are effective against both influenza A and B viruses [55–57].

The Neuraminidase-inhibitor drugs showed ineffectiveness against influenza C infection [l]. However, Baloxavir exhibits broad-spectrum antiviral activity, including influenza C and D viruses [58].

Generally, antiviral drugs are targeting the viral components that are not able to combat the genetically altered emerging strains [59, 60] so drug-resistant strains emerge. Recent studies show neuraminidase inhibitor drug Oseltamivir also generates resistant viruses [17] like the M2-inhibitor drugs. Excessive dosages of the antiviral drugs [61] and selection pressure due to global Oseltamivir administration [62] unlikely contribute to the emergence of drug-resisting viruses.

### Need for new remedy

The drawback in vaccine preparation and conventional therapies makes scientists turn their attention to powerful herbal medicines that are in great demand in the developing world [63]. A substantial increase in the consumption of herbal medicines for chronic diseases like influenza is growing exponentially [64, 65]. They are the promising alternatives reckoned for their safety, compatibility, efficacy, and minimum side effects [66]. Hence, in this review, we highlight the potent herb exhibits anti-influenza activities for the control and management of influenza.

## Methods

An intense database search conduct by using the keywords like herbs used against influenza, traditional medicines for influenza, and ethno medicines for influenza. Through this, we get the relevant literature up to October 2020 from scientific databases like Google Scholar, Springer, Elsevier, Sage, Taylor & Francis, Hindawi, Wiley, Research Gate, PubMed, Scopus, Web of Science, and Shodhganga. The required data were obtained from both research and review articles published in national and international reputed journals.

## Alternate therapies

Complementary and traditional medicines have been utilized for several years in various parts of the world to reduce human diseases [67]. For all these medicines, plants are the richest source. They have the active constituent phytochemical metabolites produced by the process called metabolism. This metabolism is categorized into two types as primary and secondary. Table 1 is a list of various herbs with phytochemical constituents with anti-influenza activity.

**Table 1:** List of herbs and their phytochemical constituents active against influenza

S. no	Herbal plant	Family	Parts of plant	Phytochemical constituents or compounds showing anti-influenza activity	Activity	Influenza virus type	Strain	Reference
1	*Albizia julibrissin*	Leguminosae	Dry stem bark	Saponin: AJS75	i. Potent adjuvant ii. Induce cellular and humoral response iii. Stimulate cytokines and chemokines	Avian influenza	Recombinant fowl pox virus vector–based avian influenza vaccine (rFPV)	[68]
2	*Aloe vera*	Asphodelaceae	Roots	Anthraquinones:3-(2´,3´,4´,6´-Tetra-O-acetyl-β-d-glucopyranosyl-aloesaponarin-I (5) and 3-(2´,3´,4´,6´-Tetra-O-acetyl-β-d-glucopyranosyl- aloesaponarin-II (7)	Inhibit virus replication	Influenza A H1N1	A/Yucatán/2370/09, A/Mexico/InDRE797/10 (H1N1)	[69]
3	*Alpinia officinarum*	Zingiberaceae	Rhizomes	Diarylheptanoids: 7-4″-hydroxy-3″-methoxyphenyl)-1-phenyl-4E-hepen-3-one (3) and (5S)-5-hydroxy-7-(4″-hydroxyphenyl)-1-phenyl-3-heptanone (8)	Potent anti-influenza activity	Influenza A H1N1	A/PR/8/34( H1N1)	[70]
				Diarylheptanoids: 7-4″-hydroxy-3″-methoxyphenyl)-1-phenyl-4E-hepen-3-one (AO-0002) and (5S)-5-hydroxy-7-(4″-hydroxyphenyl)-1-phenyl-3-heptanone (AO-0011)	AO-0002: i. Suppress the expression of viral antigen and mRNA synthesis ii.Reduce the bodyweight loss and iii. Enhance the survival period of infected mice, iv.Reduces the virus titer in lungs BALF	Influenza A H1N1, H3N2 and B	A/PR/8/34, oseltamivir-resistant A/PR/8/34, A/Bangkok/93/03 ( H1N1), A/Ishikawa/7/82, A/Fukushima/13/43 ( H3N2) and B/Singapore/222/79, B/Fukushima/15/93 (B)	[71]
4	*Andrographis paniculata*	Acanthaceae	Not mentioned	Diterpenoid: 14-a-lipoyl andrographolide (AL-1),14-deoxy-11,12-dehydroandrographolide (DAP)	AL-1: i. HA inhibition ii. Prevents virus adsorption iii. Increase survivality of infected mice	Influenza A H9N2, H5N1, H1N1	A/Chicken/Guangdong/96 (H9N2), A/Duck/Guangdong/99 (H5N1), and A/PR/8/34 (H1N1))	[72]
					DAP: i. Inhibits viral progeny ii. Inhibit viral nucleoprotein (NP) mRNA, NP, and NS1proteins iii. Inhibit nuclear export of viral ribonucleoprotein (vRNP) complexes iv. Reduction of proinflammatory cytokines and chemokines	Influenza A H5N1 H1N1,H3N2	A/chicken/Hubei/327/2004, A/duck/Hubei/XN/2007 (H5N1), A/PR/8/34, A/NanChang/08/2010 (H1N1), and A/HuNan/01/2014 (H3N2)	[73]
5	*Arctium lappa*	Compositae	Fruits	Liganan: Arctiin and arctigenin	Arctiin: i. Enhance virus specific antibody production ii. Reduce virus yield along with oselatamivir Arctigenin: i. Interact with the early stage of viral replication but not inhibit its cellular penetration, ii. Inhibits viral progeny and release	Influenza A H1N1	A/NWS/33( H1N1)	[74]
6	*Aronia melanocarpa*	Rosaceae	Fruits	Phenolic acid: Ellagic acid Flavone: Myricetin	Araonia extract: HA inhibitors; Ellagic acid: Increase the survival rate by 37.5% of rPR8-GFP virus–infected mice; Myricetin: Provide 50% survival rate of rPR8-GFP virus–infected mice	Influenza A H1N1, H3N2, recombinant H1N1, and influenza B	A/Korea/01/2009, A/Korea/2785/2009(H1N1), A/Perth/16/2009(H3N2), B/Brisbane/60/2008(B) and A/Puerto Rico/8/34(recombinant H1/PR8 expressing green fluorescent protein (rPR8-GFP)	[75]
7	*Astragalus sp*	Fabaceae	Not mentioned	Polysaccharide: *Astragalus* polysaccharide (APS)	APS: i. Stimulation of CEF Proliferation ii. Pre-addition (321.25μg/mL), post-addition, and simultaneous addition shows virus reduction iii. Upregulated IL-4, IL-10, LITAF, and IL-12 cytokine expression iv. CD3+, CD4+, and CD8+ T cell surface markers were increased	Influenza A H9N2	Not mentioned	[76]
8	*Azadirachta indica*	Meliaceae	Leaf	Flavonols: Hyperoside	Show best interactions with conserved residues of nucleoprotein	Influenza A H1N1 ( PDB ID:3RO5)	A/Wilson-Smith/1933	[77]
9	*Bupleurum chinense*	Apiaceae	Aerial part and root	Polysaccharide: Bupleurum chinense polysaccharide (BCPS)	BCPS: Immunostimulating agent enhance antibody	Influenza virus	Not mentioned	[78]
10	*Caesalpinia sappan*	Leguminosae	Dried heartwood	Chalcone: 3-deoxysappanchalcone, and sappanchalcone Homoisoflavonoids: Sappanone A, Brazilin	Chalcone: Show high inhibition against H3N2	Influenza A H1N1, H3N2 and B	A/PR/8/34 (H1N1), A/Guangdong/243/72 (H3N2), and B/Jiangsu/10/2003	[79]
					Homoisoflavonoids: Contain α, β-unsaturated carbonyl group in A-ring critical role in NA inhibition	Influenza A H1N1, H3N2,H9N2	A/PR/8/34(H1N1), A/Hong Kong/8/68 (H3N2), and A/Chicken/Korea/MS96/96 (H9N2)	[80]
11	*Camellia sinensis*	Theaceae	Leaves	Amino acid: Theanine Flavon-3-ol: (-) epigallocatechin (EGC), (−)-epigallocatechin gallate (EGCG), (−)-epicatechin gallate (ECG), EGCG-C-16, (−) Epigallocatechin gallate (EGCg) and theaflavin digallate (TF3), Theaflavin, Tannin: Strictinin	Theanine and Catechin: Effective prophylaxis	Not mentioned	Not mentioned	[81]
					EGC: Acidification of ELS inhibition	Influenza A H1N1, H3N2 and B	A/PR/8/34 (H1N1),A/Aichi/2/68(H3N2), B/Singapore/222 Sing (B)	[82]
					EGCG,ECG: HI, NI activity and suppression of viral RNA synthesis	Influenza A H1N1, H3N2 and B	Influenza A/Chile/1/83( HIN1), A/Sydney/5/97 (H3N2) and B/Yamagata/16/88 (B)	[83]
					EGCG-C-16: Potent infection inhibitor	Influenza A H1N1, H3N2,H5N2, and B	A/Puerto Rico/8/34, A/Beijing/262/95, Yokohama/77/2008,Yokohama/63/2007,A/Yokohama/91/2008(H1N1), A/Panama/2007/99 (H3N2), A/Duck/HongKong/342/78 (H5N2) and B/Yamanashi/166/98 (B)	[84]
					EGCg, TF3: Virus agglutination, prevent adsorption, HA inhibition	Influenza A H1N1 and B	A/Yamagata/120/86( H1N1), B/USSR/100/83 (B)	[85]
					Theaflavin: Potent natural inhibitor	Influenza A H1N1 NA	Predicted structure for in silico study	[86]
					Strictinin: i.Acts directly with the viral particles ii. Inhibit the early stage of viral entry and virus-induced hemifusion	Influenza A H1N1,H3N2,H5N3, and B	A/PuertoRico/8/34 , A/WSN/33 (H1N1), A/Memphis/1/71, A/Aichi/2/68, A/swine/Hokkaido/10/85 (H3N2), A/duck/HK/313/4/78 (H5N3), and B/Lee/40 (B)	[87]
12	*Chaenomeles speciosa*	Rosaceae	Dried fruit	*Benzoic acid derivative: 3,4-dihydroxybenzoic acid, flavonol: quercetin, ester: methyl 3-hydroxybutanedioic ester*	*i. Inhibits TNF-α production* *ii. 3, 4-dihydroxybenzoic acid and quercetin*: Dose dependent DPPH radical scavenging activity iii. *methyl 3-hydroxybutanedioic ester: IL-6 production inhibition* *iv. 3, 4-dihydroxybenzoic acid and methyl 3-hydroxybutanedioic ester : NA inhibition*	Influenza A H1N1	H1N1 (*A/PR/8/34)*	[88]
13	*Cinnamomum cassia*	Lauraceae	Cortex	Aldehyde: Trans cinnamaldehyde (CA) Coumarin: 7-hydroxycoumarin (7HC) Cinnamyl derivatives: 4-allylanisole, cinnamic acid ethylester, acetic acid cinnamylester, 2X-hydroxyacetophenone, and 2-hydroxycinnamic acid	Aldehyde: i. Dose-dependent inhibition of virus ii. Affects protein synthesis at post-transcriptional level iii. Increase survival rate with reduced viral titer in virus-infected mice	Influenza A H1N1, H3N2, and B	H1N1 (A/PR/8/34, A/USSR/92/77), H3N2 (A/Aichi/2/68), and B (B /Lee/40)	[89]
					Cinnamyl derivatives: Anti-pyretic, suppress the rise of interleukin-1α production	Influenza A H1N1	A/PR/8/34( H1N1)	[90]
					7HC: Anti-pyretic, suppression of pro-inflammatory cytokine,interleukin-1α and Th1 cytokine (IL-12 and interferon-gamma), Reduce virus load in BALF	Influenza A H1N1	A/PR/8/34( H1N1)	[91]
14	*Curcuma longa*	Zingiberaceae	Rhizome	Sesquiterpenoids: germacrone Diarylheptanoids: curcumin, curcuminoids	Germacrone: Reduction of viral protein expression, RNA synthesis, and the progeny viruses	Influenza A H1N1, H3N2 and B	A/PuertoRico/8/34, A/human/Hubei/1/2009, A/human/WSN/33 (H1N1), A/human/Hubei/3/2005 (H3N2), and B/human/Hubei/1/2007 (B)	[92]
					Curcumin: HA inhibitors	Influenza A H1N1, H6N1	A/Puerto Rico/8/34( H1N1), A/chicken/Taiwan/NCHU0507/99( H6N1)	[93]
					Curcuminoids: NA inhibitors	Influenza A H1N1,H9N2	A/California/08/2009, A/Sw/Kor/CAH1/04, H274Y mutant (H1N1) A/Chicken/Korea/O1310/2001 (H9N2)	[94]
15	*Dendrobium nobile*	Orchidaceae	Stem	Alkaloid: dendrobine	Inhibit early stage of viral replication, binding with the viral NP suppress its export, deactivation vRNP complex	Influenza A H1N1, H3N2	A/FM-1/1/47, A/Puerto Rico/8/34 H274Y ( H1N1) and A/Aichi/2/68 ( H3N2)	[95]
16	*Elsholtzia rugulosa*	Lamiaceae	Whole plants	Flavonoids: Apigenin and Luteolin	Exhibited the highest NA inhibition against H3N2.	Influenza A H1N1, H3N2, and B	H1N1 (A/PR/8/34), H3N2 (A/Jinan/15/90), and B (B/Jiangsu/10/2003)	[96]
17	*Ephedra sinica*	Ephedraceae	Whole plant	Flavan-3-ol: (+)-catechin	Acidification of ELS, Dose-dependent inhibition of virus growth	Influenza A H1N1	APR/8/34(H1N1)	[97]
18	*Ginkgo biloba*	Ginkgoaceae	Leaf	Biflavonoid: Ginkgetin, and its conjugates (6R, 6S, 7R, 7S, 8R, 8S, 9R, 9S)	8R and 8S: Low cytotoxic effect, high sialidase activity with increased survival rate	Influenza A H1N1, H3N2 and B	A/PR/8/34 (H1N1), A/Guizhou/54/89 (H3N2), B/Ibaraki/2/85 (B)	[98]
19	*Glycyrrhiza inflata*	Fabaceae	Root	Chalcone: Echinantin and isoliquiritigenin	Echinantin and Isoliquiritigenin without prenyl group: Strong NA inhibitor Echinantin: Synergistic effects against NA of H274Y virus	Influenza A H1N1, H9N2, H1N1 (WT), and H1N1 (H274Y))	Not mentioned	[99]
20	*Glycyrrhiza uralensis*	Fabaceae	Root	Chalcone: Isoliquiritigenin	Strong NA inhibitor	Influenza A H1N1	rvH1N1 (A/Bervig_Mission/1/18)	[100]
21	*Hamamelis virginiana*	Hamamelidaceae	Bark	Phenolic acid: Gallic acid, hamamelitannin Tannin: Tannic acid, and pentagalloylglucose Catechin: epigallocatechin gallate	Gallic acid, Epigallocatechin gallate, Hamamelitannin :NA inhibition Tannic acid: Inhibition of viral binding and neuraminidase	Influenza A H1N1, H3N2, H7N9	A/Puerto Rico/8/34, A/Luxembourg/46/2009 (H1N1), A/Luxembourg/01/2005 ( H3N2), A/Anhui/01/2013 ( H7N9)	[101]
22	*Houttuynia cordata*	Saururaceae	Aerial parts, Whole plant	Aldehyde: Lauryl aldehyde, and Capryl aldehyde Ketone: Methyl n-nonyl ketone	Exerts virucidal activity by interfere with viral envelope	Influenza A H1N1	NWS	[102]
				Polysaccharide: *Houttuynia cordata* polysaccharide [HCP]	HCP: i. Inhibits pulmonary inflammatory cytokine and expression of TLR4-NF-κB ii. Lessens the severity of organ injury	Influenza A H1N1	A/FM/1/47( H1N1)	[103]
				Flavone: Quercetin 3-rhamnoside (Q3R)	Reduction in viral mRNA synthesis, Inhibit initial stage of replication	Influenza A H1N1	A/WS/33 (H1N1)	[104]
23	*Isatis indigotica*	Brassicaceae	Root	Polysaccharide: *Isatis indigotica* root polysaccharide (IRPS), Alkaloid: Indirubin, Lignan: Clemastanin	IRPS: i. Inhibit RNA and Protein synthesis ii. Induce cytokine production iii. Reduce pulmonary inflammation	Influenza A H3N2	A/swine/Henan/2010 (H3N2)	[105]
					Indirubin: Inhibition of RANTES in virus-infected H292 cells. Interrupts virus-induced NF-κB translocation, p38 MAP kinase activation.	Influenza A H1N1	A/NWS/33 (H1N1)	[106]
					Clemastanin: i. Early stage of replication ii. Interfere with RNP export iii. Not create drug resistance	Influenza A H1N1	A/PR/8/34 (H1N1)	[107]
24	*Lycoris radiate*	Amaryllidaceae	Bulb	Alkaloid: Lycorine and Hemanthamine	Block vRNP nuclear export, Resist pro-apoptotic stimuli and cytoskeleton disruption	Influenza A H5N1, H3N2, H1N1, H9N2	A /Chicken/GuangDong /178 /2004, A/CK/GD/178/04, A/DK/GD/212/04 (H5N1), A/Swine/GD/166/06 (H3N2), A/CK/HN/170/03, A/PuertoRico/8/34 (H1N1), A/CK/GD/400/07, A/CK/GD/228/04 (H9N2)	[108, 109]
25	*Narcissus tazetta*	Amaryllidaceae	Bulbs	Proteins: Narcissus tazetta lectin [NTL], NTP	NTL: i.Inhibit early phase of replication ii. Interacting with surface glycoproteins iii. Avoid the virus adherence and fusion	Influenza A H1N1, H3N2,H5N1 and B	A/HongKong/CUHK-13003/2002 (H1N1), A/HongKong/CUHK 22910/2004 (H3N2), A/HongKong/483/1997 (H5N1), and B/HongKong/CUHK-24964/2004 (B)	[110, 111]
					NTP: NA inhibitor	Influenza A H1N1	Not mentioned	[112]
26	*Olea eurolaea*	Oleaceae	Leaves and fruits	**Catechol:** Hydroxytyrosol (HT)	i. Inactivates the viruses ii. Affects NP protein synthesis iii. Suppresses mRNA synthesis iv. Structural disruption	Influenza A H1N1, H3N2, H5N1, H9N2	A/Hokkaido/30/2000 (H1N1), A/Hokkaido/52/98 (H3N2), A/chicken/Yamaguchi/7/04 (H5N1), and A/chicken/Yokohama/aq55/01 (H9N2)	[113]
27	*Panax ginseng*	Araliaceae	Not mentioned	Polysaccharide: *Panax ginseng* polysaccharide [GP] Glycosides: Ginsenoside PPT, Re	GP: i. Reduce inflammatory cytokine (IL-6) ii. regular consumption protects against heterosubtypic lethal challenges	Influenza A H1N1,H3N2	A/PR/8/34 ,A/California/04/2009 (H1N1) and A/Philippines/82 ( H3N2)	[114]
					PPT: i. Inhibits IP-10 production ii. Regulating the micro RNA, miR-15b Re: i. Partially reduced the virus-adapted apoptosis ii. Provide cytoprotection iii. Increase cell viability effect	Influenza A H9N2	A/Quail/Hong Kong/G1/97 (H9N2)	[115]
28	*Panax quinquefolium*	Araliaceae	Not mentioned	Glycosides: Ginsenoside	i. Prevent lethal lung damage ii. Interact with HA of virus iii. Prevents virus attachment with α 2–3′ sialic acid receptors	Influenza A H1N1	A/Nanchang/8002/2009 H1N1 (NC2) (H1N1)	[116]
29	*Pandanus amaryllifolius*	Pandanaceae	Leaves	Proteins: *Pandanus amaryllifolius* (PYM2) Lectin: Pandanin	Pandanin: Hemagglutinator	Influenza A H1N1	Not mention	[117]
					PYM2: Upregulation of cytokines IL-1β, IL-12, IFN-γ, and TNF-α	Influenza A H1N1, H3N2, H5N1, and B	Not mention	[110]
30	*Perilla frutescens*	Lamiaceae	Seeds	Flavones: Luteolin Phenolic acid: Rosmarinic acid	NA inhibitor	Influenza A H1N1 NA	Recombinant virus H1N1 neuraminidase (rvH1N1 NA E.C. 3.2.1.18)	[118]
31	*Pithecellobium clypearia*	Fabaceae	Leaves and twigs	Flavan-3-ol: (2R,3R)-7-O-galloylplumbocatechin A (1), (−)-5,3′,4′,5′-tetrahydroxyflavan-7-gallate (2), (+)-3,5,3′,4′,5′-penta-hydroxyflavan-7-gallate (3), and (−)-7,4′-di-O-galloyltricetiflavan (4), gallocatechin-7-gallate (J10688)	Flavan-3-ol compounds 1–4: Inhibit the expression of proinflammatory cytokines IL-6 or MCP-1 Compound 1 and 2: Moderate NA activity	Influenza A H1N1, H3N2, and B	A/PR/8/34 (H1N1), A/Sydney/5/97 (H3N2), B/Jiangsu/10/2003 (B)	[119]
					J10688: i. Effective CLK1 inhibitor regulates M2 alternative splicing ii.Inhibit pro-inflammatory cytokines IFN-γ, IL-6, TNF-α, and IL-1β iii. Increase survival rate by prevent viral infection and lung injury iv. Decrease viral NP, M2, and RNA synthesis	Influenza A H1N1, H3N2 and B	A/PR/8/34 (H1N1), A/Sydney/5/97 (H3N2), and B/Jiangsu/10/2003	[120]
32	*Pogostemon cablin*	Lamiaceae	Dried aerial part	Sesquiterpene: Patchouli alcohol	i. Target virus particles and cellular PI3K/Akt and ERK/MAPK signaling pathways ii. Reduction of a viral nucleoprotein iii. Increased survival and decreased pneumonia symptoms in virus-infected mice iv. Reduced viral multiplication	Influenza A H1N1	Influenza A viruses H1N1 (A/Puerto Rico/8/34), H1N1 (A/NWS/33), and H1N1 (A/Virginia/ATCC1/2009)	[121]
					i. Elevation of IgA, IgM, and IgG antibodies ii. Increased CD3+ and CD4+ T cell levels iii. Reduce lung inflammation by secreting anti-inflammatory cytokines IL-10 and IFN-γ	Influenza A H1N1	A/FM/1/47(H1N1)	[122]
					i. Inhibit viral penetration ii. Protect the infected mice from lethal effect iii. Perfect binding with virus NA	Influenza A H2N2	A/Leningrad/134/17/1957(H2N2)	[123]
33	*Polygonatum odoratum*	Asparagaceae	Rhizomes	Proteins: Polygonatum odoratum Lectin (POL)	POL: i. Antiviral activity against H1N1 and H5N1 ii. Produce immunomodulatory effects by upregulation of several cytokines like IL-1β, IL-12p35, IFN-γ, and TNF-α Lectin: Antiviral activity against H1N1 and H5N1	Influenza A H1N1, H3N2,H5N1 and B	Not mention	[110]
34	*Polygonum cuspidatum*	Polygonaceae	Rhizome, dried roots	Stilbenes: Resveratrol, (E)-3, 5, 12-trihydroxystilbene-3-O-beta-d-glucopyranoside-2′-(3″, 4″, 5″-trihydroxybenzoate) Flavan-3-ol: Catechin-3-O-gallate	Virus and neuraminidase inhibitor	Influenza A H1N1	Not mentioned	[124]
35	*Psoralea carylifolia*	Fabaceae	Seeds	Meroterpene: (+)-(*S*)-bakuchiol, and its enantiomer, (−)-(*R*)-bakuchiol	(+)-(S)-bakuchiol: i. Inhibited the H1N1 strains ii. Decreased viral mRNAs and protein expression iii. Induce Nrf2 activation and upregulated *NQO1*, p53 and *GSTA3* mRNA levels thereby inhibit viral growth	Influenza A H1N1, H3N2	A/PR/8/34, A/CA/7/09 (H1N1), and A/Aichi/2/68 (H3N2)	[125]
36	Punica granatum	Lythraceae	Fruit	Hydrolysable tannin: punicalagin	i. Produce direct inhibitory and virucidal effect. ii. Affect viral replication by target its attachment	Influenza A H1N1, H3N2, and influenza B	A/USSR/90/77 (H1N1), A/Hong Kong/2/68, A/HK (H3N2), B/Harbin/07/94 (B)	[126]
37	*Rhodiola rosea*	Crassulaceae	Dried Roots	Flavonols: Gossypetin, kaempferol	NI inhibition with Potent anti-influenza activity	Influenza A H1N1,H9N2	A/PR/8/34 (H1N1) A/Chicken/Korea/MS96/96 (H9N2)	[127]
38	*Ribes nigrum*	Grossulariaceae	Fruits	Crude extracts, anthocyanin	Extract inhibits the virus release and suppress late stage of growth	Influenza A H1N1 and B	A/PR/8/34 (H1N1), B/Gifu/2/73 (B)	[128]
					Anthocyanin: Responsible for anti-influenza activity in crude extract Fractions D′ to G′: Potent against influenza A and B E′ and F′: Show Additive antiviral effects F′: Inhibit virus adsorption and release	Influenza A H1N1 and B	A/PR/8/34 (H1N1), B/Gifu/2/73 (B)	[129]
39	*Sambucus nigra*	Adoxaceae	Fruits (Berries)	Flavone: 5,7,3′,4′-tetra-O-methylquercetin (1) and 5,7-dihydroxy-4-oxo-2-(3,4,5-trihydroxyphenyl) chroman-3-yl-3,4,5-trihydroxycyclohexanecarboxylate (2)	i. Virion binding ii. Prevent host cell entry and/or recognition	Influenza A H1N1	A/PR/8/34 (H1N1)	[130]
40	*Schefflera heptaphylla*	Araliaceae	Leaf stalk	Triterpenoids: 3α-hydroxylup-20 (29)-ene-23, 28-dioic acid and 3-epi -betulinic acid 3-O-sulfate.	Inhibit virus infection	Influenza A H1N1	Not mentioned	[131]
49	*Scutellaria baicalensis*	Lamiaceae	Leaves and roots	Flavones:Baicalin, 5, 7, 4′-trihydroxy-8-methoxyflavone (F36), Isoscutellarein (5,7,8,4′-tetrahydroxyflavone), wogonin, baicalein	Baicalin: i. Neuraminidase inhibitor ii. Affects the virus budding iii. Increased survival rate	Influenza A H1N1, H3N2	i. A/FM1/1/47 (H1N1) and ii. A/Beijing/32/92 (H3N2)	[132]
					F36: i. Inhibit infection replication ii. Prevent virus proliferation iii. Reduce lung virus titers iv. Endosome/lysososme fusion inhibition	Influenza A H1N1, H3N2, and B	i. *A/PR/8/34 (H1N1)* ii. *A/Guizhou/54/89 (H3N2) and iii.B/Ibaraki/2/85 (B)*	[133, 134]
					Isoscutellarein (5,7,8,4′-tetrahydroxyflavone: i. Sialidase inhibitory effect ii. Replication inhibition	Influenza A H1N1	A/WSN/33 and A/PR/ 8/34 (H1N1)	[135, 136]
					Wogonin: i. Replication suppression ii. Induce IFN response and AMPK phosphorylation	Influenza A H1N1, seasonal H1N1, H3N2, and B of Yamagata lineage	A/Puerto-Rico/8/34(H1N1), seasonal H1N1, H3N2, and B of Yamagata lineage (Clinical influenza strains)	[137]
					Baicalein: i.Interact with NA1 active sites ii.Inhibit replication	Influenza A H1N1 (pandemic and seasonal)	A/Taiwan/CMUH/2009 (pandemic 2009 H1N1), A/Taiwan//CMUH/2007(seasonal 2007 H1N1)	[138]
42	*Solanum tuberosum*	Solanaceae	Root tuber	Anthocyanin	Additive or synergistic effect of the constituents produce antiviral effect	Influenza A H1N1 and B	H1N1 (A/PR/8/34) and B(B/Gifu/2/73)	[139]
43	*Sophora flavescens*	Leguminosae	Root, dried heart wood	Alkaloids: i. Aloperine and its derivatives ii.Matrine, Pterocarpans, Homoisoflavonoid: Sappanone A and Brazilin, Prenylflavonoid: 8-Prenylkaempferol (8-PK), Prenylflavanones	Alkaloids: i. Target NP ii. Reduce cytotoxic effect and HI inhibition similar to Oseltamivir.	Influenza A H1N1, H3N2, H9N2	A/Puerto Rico/8/34 (H1N1), oseltamivir-sensitive virus VR1679 (H3N2) A/Goose/Dalian/3/2001( H9N2)	[140, 141]
					Pterocarpan: In silico studies shows NA binding near to active site	Influenza A	PDB ID 1L7F	[142]
					Sappanone A and Brazilin: Strong NA inhibitors	Influenza A H1N1, H3N2, H9N2	A/PR/8/34 (H1N1), A/HongKong/8/68 (H3N2), a/Chicken/Korea/MS96/96 (H9N2)	[80]
					8-PK: i.Block PI3K pathway ii.Prevent NF-κB, IRF-3 activation and IκB degradation ii.Reduction of RANTES accumulation	Influenza A H1N1	A/PR/8/34 (H1N1)	[143]
					Prenylflavanones: Active against influenza infection	Influenza A H1N1	A/WSN/33/2009(H1N1)	[144]
44	*Strobilanthes cusia*	*Acanthaceae*	Not mentioned	Alkaloid: Indirubin	Inhibition of RANTES in virus-infected H292 cells Interrupts virus-induced NF-κB translocation, p38 MAP kinase activation	Influenza A H1N1	A/NWS/33 (H1N1)	[106]
45	*Taxodium distichum*	Cupressaceae	Stem	Shikimic acid	NA inhibitor	Influenza A H1N1	A/WSN/33 (H1N1)	[145]
46	*Thallasodendron ciliatum*	Cymodoceaceae	Whole plant	Ester: Asebotin Chalcone: Thalassodendrone (dihydrochalcone diglycoside)	Asebotin: Virus inhibition	Influenza A H5N1	A/chicken/Egypt/1055/2010 (H5N1)	[146]
					Inhibit virus with low cytotoxic effect	Influenza A H1N1	A virus /WSN/33 ( H1N1)	[147]
47	*Wasabi japonica*	Brassicaceae	Rhizome, fibrous root, and petiole	Isothiocyanate	Virucidal effect	Influenza virus	Not mentioned	[148]
48	*Withania somnifera*	Solanaceae	Fresh leaves	Ester: Withaferin A	High binding affinity with NA (Docking study)	Influenza A H1N1	Not mentioned	[149]
49	*Zizyphus jujuba*	Rhamnaceae	Leaves, Fruit, and dried roots	Triterpene: Betulinic acid	i. Proliferation inhibition ii. Attenuation of increased necrosis, numbers of inflammatory cells, and pulmonary edema iii. Decreases inflammatory cytokine IFN-γ	Influenza A H1N1	A/PR/8 (H1N1)	[150]
50	Not mentioned	–	–	Monoterpenes: Menthol, Eucalyptol	Strong interactions with HA (Docking study)	Influenza A H5N1	Not mentioned	[151]

### Primary metabolism

Primary metabolism is the group of all metabolic pathways synthesizing essential compounds for plant survival. Primary metabolites perform physiological functions, and these include nucleic acids, amino acids, proteins, carbohydrates, lipids, alcohol, and other natural products [152] like ester and shikimic acid.

i. Amino acid: The amino acid glutamine is essential for the synthesis of proteins and plays a critical role in the immune system. Theanine is one of the derivatives of glutamine that enhances gammadelta T cell function in influenza infection. Matsumoto et al. conducted a randomized, double-blind, placebo-controlled trial among 197 healthcare workers to evaluate the efficacy of *Camellia sinensis* Theanine and Catechin. The author divided the participants into two groups. The first group of 98 members receives catechin (378 mg) and theanine (210 mg) daily whereas the second group of 99 members act as the control group receives placebos. The main source of outcome is the incidence of clinically defined influenza infection, laboratory-confirmed influenza with viral antigen, and the time patient is free from clinically defined infection. From this study, the authors observed 4 participants in the first group and 13 from the control group report clinically defined influenza infection, whereas the laboratory-confirmed infection prevails in 1 member of the first group and 5 from the control group and they are statistically insignificant. From this, they concluded Theanine and Catechin might be an effective prophylaxis against influenza infection [81].
ii. Proteins: Lectins are the carbohydrate-binding protein noted in plants like *Pandanus amaryllifolius* [110, 117], *Narcissus tazetta*, and *Polygonatum odoratum* which exerts antiviral activity against influenza viruses [110].

Pandanin, a new antiviral lectin protein, shows its antiviral activity against the H1N1 virus at EC_50_ of 15.63 μM with hemagglutinating activity [117]*.*

Ooi VEC et al. screened 10 purified herbal compounds from 30 herbal extracts and perform antiviral potential studies. From the 10 herbal compounds, three proteins PYM2 of *Pandanus amaryllifolius*, NTL of *Narcissus tazetta*, and POL of *Polygonatum odoratum* exhibit antiviral infectivity against H1N1, H3N2, H5N1, and influenza B viruses, observed through plaque reduction assay. AntiH5N1 efficacy of PYM2 (IC_50_ 26.03μg/mL) and POL (IC_50_ 6.23μg/mL) are further investigated by introducing 5 mg/kg of the proteins in mouse macrophages where the upregulation of cytokines IL-1β, IL-12, IFN-γ, and TNF-α was observed [110].

*Narcissus tazetta* lectin is a mannose-binding Lectin protein. Linda S M Ooi et al. describe NTL viral inhibition activity by using an extracellular virus yield reduction assay. NTL inhibits H1N1, H3N2, and influenza B viruses (A(H1N1)/HongKong/CUHK-13003/2002, A(H3N2)/HongKong/CUHK22910/2004, A(H5N1)/HongKong/483/1997, and B/HongKong/CUHK-24964/2004) in a dose-dependent manner (EC_50_ values ranging from 0.02 μg/mL to 1.33 μg/mL) but with moderate inhibition on H5N1. The study also shows evidence of NTL inhibiting the H1N1’s early phase of replication by interacting with surface glycoproteins, thereby avoid the virus for adherence and fusion [111].

NTP is a fetuin-binding non-specific lipid transfer protein (nsLTPs) of *Narcissus tazetta* that blocks the neuraminidase of H1N1 (EC_50_ of 4.47 mg/mL) thereby inhibiting the replication which is revealed from a MTT assay [112].

iii. Carbohydrates: Carbohydrates are the major constituents of the plant which are produced by the photosynthetic process [153]. They are produced in larger amounts and are classified into four types as Monosaccharides, Disaccharides, Oligosaccharides, and Polysaccharides.

Monosaccharides are the simplest carbohydrates that have polyhydroxy aldehyde or ketone groups [154]. Hayashi et al. demonstrate direct influenza H1N1 inhibitory activity of the *Houttuynia cordata* steam distillate without any cytotoxic effect against HeLa and MDCK cells. Aldehyde such as lauryl aldehyde and capryl aldehyde and ketone including methyl-nonyl ketone were the components of distillate which inactivate the influenza virus. The virus inactivation assay reveals either the distillate or its components exert virucidal activity by interfering with the function of the viral envelope [102].

Hayashi et al. isolated trans cinnamaldehyde (CA) from the cortex of *Cinnamomum cassia* and analyze its anti-influenza efficiency against H1N1 (A/PR/8/34, A/USSR/92/77), H3N2 (A/Aichi/2/68), and B (B /Lee/40). The in vitro study reveals dose-dependent inhibition of CA where 40μM reduces 29.7% virus yield at 3 h p.i. and 200 μM treatment does not show the virus growth.CA affects protein synthesis at post-transcriptional level but not degrade the preexisting or synthesized proteins. To the mouse lung-adapted PR8 virus–infected mice, CA administered through inhalation and nasal inoculation which shows 100% and 70% increasing survival rate on 8 days without remarkable weight loss. Notably, CA inhalator mouse bronchoalveolar lavage fluid (BALF) shows a decrease in virus yield by 1 log [89].

Polysaccharides are complex monosaccharides that play an essential role in the pharmaceutical industry for their effective inhibitory response against viruses like Influenza, HIV, Coxsackie virus B3 [CVB3], herpes simplex virus, hepatitis virus, and cytomeglalovirus [155]. Various polysaccharides isolated from the herbs like *Bupleurum chinense* [BCPS], *Houttuynia cordata* [HCP], *Isatis indigotica* root [IRPS], *Panax ginseng* [GP], and *Astragalus* polysaccharide (APS) proved to be boosting up various immunological activities and prevent the effect of influenza infection.

*Bupleurum chinense* polysaccharides [BCPS] act as an immunostimulating agent which enhances anti-influenza virus antibody. This was well demonstrated by Zhang and Chen’s studies, by injecting 100 mg/kg of BCPS in normal and influenza-infected mice. The results show increased lymphocyte transformation and enhanced NK cell function [78].

*Houttuynia cordata* polysaccharide [HCP] lessens the severity of organ injury due to influenza virus infection. Zhu et al. discovered its role by administered orally by gavages to H1N1 (A/FM/1/47)-infected mice and observed an increase in survival rate through inhibition of the release of pulmonary inflammatory cytokine and expression of TLR4-NF-κB. Restoring and improvement of damaged tissue also noted [103]*.*

*Isatis indigotica* root polysaccharide (IRPS) show a low cytotoxic effect and promote proliferation (1.25 mg/mL concentration) in MDCK cells infected with swine influenza virus (SIV) H3N2 of the strain (A/swine/Henan/2010). Through MTT assay, the preventive, inhibitory, and direct effect of IRPS against SIV was observed. IRPS (312.5–1250 μg/mL) increases the percentage of prevention from 12.64 to 74.00% which shows the MDCK cell protection with enhanced antiviral ability. In addition to that, the cell membrane was stabilized by IRPS, which provides the potential to inhibit adsorption, penetration, and infection of SIV. A dose-dependent increase of IRPS (156.25–1250 μg/mL) increased the percentage of inhibition from 31.92% to 84.95% suggested; IRPS may play a potent role in inhibiting viral RNA and protein synthesis. Surprisingly, IRPS direct effect was found to be weak in SIV-adapted MDCK cells. By in vivo study, the mice were orally administered with varying doses of IRPS chosen as 75 mg/kg/day, 50 mg/kg/day, and 25 mg/kg/day before the infection. The mice at 3 dpi show significant clinical signs, while at high dosage applied, 5 dpi regained the weight. The IRPS reduced pathological changes, with improved lesions and normal alveolar structure. Host immunity signaling molecule NO (Nitric oxide) was increased at 2 and 5 dpi; similarly, the antibody IgG in blood serum is increased significantly at 9 dpi. IRPS induces cytokine production and reduces pulmonary inflammation, which was evident from the detection of cytokine in lung homogenate [105].

*Panax ginseng* polysaccharide [GP] increases the survival rate of H1N1 (A/PR/8/34)-, (A/California/04/2009)-, and H3N2 (A/Philippines/82)-infected mice by providing intranasal 25 mg/kg or intravenous 10 mg/kg doses. The GP-treated H1N1 infect mice exhibit a lower level of viral titer along with reduced inflammatory cytokine (IL-6). It is noted that a higher dosage does not increase the survival rate. GP provides protection effect even before the influenza infection, and regular consumption protects against heterosubtypic lethal challenges [114].

In this study, Kallon et al. use *Astragalus* polysaccharide (APS) against H9N2-infected chick embryo fibroblasts (CEF) culture. APS of 321.25 μg/mL concentrations is determined as optimal for the stimulation of CEF Proliferation. Appropriate dosage of APS administered during pre-addition (321.25 μg/mL), post-addition, and simultaneous addition shows a significant reduction of viruses. APS upregulated IL-4, IL-10, LITAF, and IL-12 cytokine expression but IFN-γ, LITAF, IL-6, and IL-12 were downregulated. APS inoculation decreased MHC I and MHC II expression but increased after H9N2 infection. Peripheral blood lymphocytes expressing CD3+, CD4+, and CD8+ T cell surface markers were increased after APS immunization. Post-immunization of APS (5mg/kg) enhanced the antibody titer at 7 and 14 days [76].

iv. Ester: Ester has ideal characteristics with chemical stability. Its unique characters attract to create prodrug [156]. Several ester compounds show promising anti-influenza features.

Zhang et al. isolated 13 compounds from the dried fruit powders of *Chaenomeles speciosa* and identify 3, 4-dihydroxybenzoic acid (Benzoic acid derivative), quercetin (flavonol), and methyl 3-hydroxybutanedioic ester (Ester) showed remarkable anti-influenza activity*.* The compounds 3, 4-dihydroxybenzoic acid (IC_50_ 1.02 μg/mL), and quercetin (IC_50_ 3.82 μg/mL) displayed significant dose-dependent DPPH radical scavenging activity*.* 5μg/mL of 3, 4-dihydroxybenzoic acid, quercetin, and methyl 3-hydroxybutanedioic ester inhibit TNF-α *by* 22.73%, 33.14%, and 37.19% and NO production inhibition. High IL-6 production inhibition (39.79%) was observed in methyl 3-hydroxybutanedioic ester. The compounds 3, 4-dihydroxybenzoic acid (IC_50_ 1.27 μg/mL) and methyl 3-hydroxybutanedioic ester (IC_50_ 1.90 μg/mL) featured H1N1 (A/PR/8/34) NA inhibition activity *i*n a dose-dependent manner [88].

The study of Ibrahim et al. informed that a new diglyceride ester along with asebotin (a dihydrochalcone) from *Thallasodendron ciliatum* exerted anti-AIV activity against A/chicken/Egypt/1055/2010 (H5N1) in MDCK cells. This compound inhibited the virus with a concentration of 1 ng mL^−1^ by a percentage of 67.26 and 53.81. Its efficacy was similar to zanamivir which showed a higher concentration of inhibition of 10 ng mL^−^ 1[146].

Cai et al. validated Withaferin A of *Withania somnifera* as a potent source for H1N1 NA inhibition through docking studies. In the study, Withaferin A shows a high binding affinity towards NA [149].

xxii. Shikimic acid: Shikimic acid is an important biochemical metabolite and its pathway is essential for the synthesis of plant amino acid [157]. Antiviral drug Tamiflu® is prepared by using shikimic acid as base material [158]. Several plants and their parts like fruits of *Illicium religiosum* and *Illicium verum*, barks of *Illicium anisatum*, *Pinus thunbergii*, and *Pinus densiflora*, stems and leaves of *Saxifraga stolonifera*, and *Houttuynia cordata* exhibits high shikimic acid content [159].

Recently shikimic acid has been noted in *Taxodium distichum* extract and it is a carbocyclic sialic acid analog. Hence, the authors Hsieh et al. [145] suggest that it may also exert the function as a neuraminidase inhibitor.

### Secondary metabolism

**Fig. 3:**
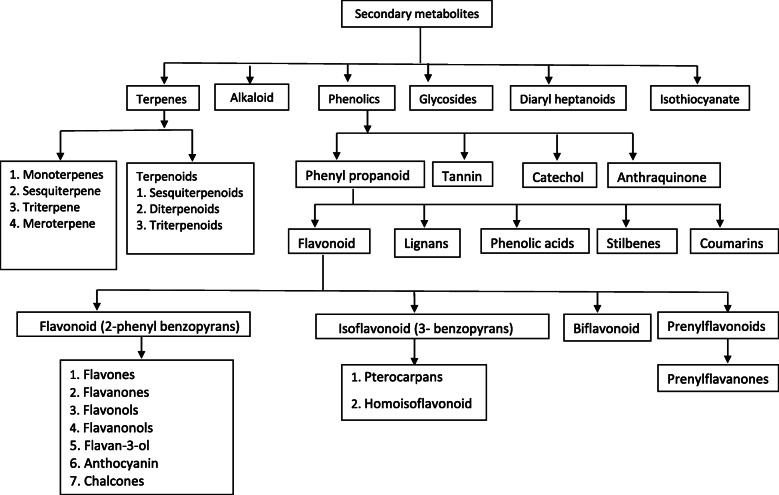
Classes of secondary metabolites

Secondary metabolism comprises metabolic pathways which are not essential for the functioning or survival of the plant. Both primary and secondary metabolism, along with their regulatory pathways facilitates the plant to survive under stressful conditions [160]. Interestingly the secondary metabolites are derived from the primary metabolites and their various pathways [161]. Indeed, plants produce a wide array of secondary metabolites, but there is no precontrived and commonly agreed system of classification [162]. However, based on their chemical structure and biosynthetic origin, they are classified into three major classes as terpenes, alkaloids, and phenolics [163]. In addition to this, some special categories like terpenoids, glycosides, diaryl heptanoids, and isothiocyanates are included under secondary metabolites. Figure 3 categorizes the various classes of secondary metabolites.

#### Terpenes

Terpenes are a large class of secondary metabolites and they are classified based on the isoprene unit. monoterpenes, sesquiterpene, triterpene, and meroterpene are the various classes of terpenes [164, 165].

#### Monoterpenes

Gangopadhyay et al. used Hex 5.1 Docking software and dock 13 herbal and lead inhibitors with wild-type and mutant haemagglutinins of H5N1. The study reveals that the monoterpenes menthol and eucalyptol show strong interactions by binding to glutamate 11 site of haemagglutinin thereby inhibiting viral fusion with the host cell [151].

#### Sesquiterpene

Patchouli alcohol (PA) is a sesquiterpene extracted from *Pogostemon cablin*. Yu et al. investigated both in vitro and in vivo anti-influenza activity of PA against H1N1 (A/Puerto Rico/8/34, A/NWS/33, and A/Virginia/ATCC1/2009). The in vitro time-of-addition assay (TOA) indicates the reduction of viral multiplication is observed before and after infection. The anti-viral mechanisms of action were studied, by using neuraminidase inhibition assay (NI), hemagglutination inhibition (HI) assay, and western blot assay. This study explores PA neither inhibits NA nor HA of the influenza viruses, but it targets virus particles and cellular PI3K/Akt and ERK/MAPK signaling pathways thereby arresting the viral infection. Reduction of a viral nucleoprotein by PA was noticed by western blot assay. The in vivo study is carried by intranasal administration of PA to the IAV-infected mouse pneumonia model. This study explores the increased survival, decreased pneumonia symptoms comparable to the effects of Oseltamivir [121].

Li et al. perform in vivo anti-influenza studies by oral administration of Patchouli alcohol (PA) (20 mg/kg to 80 mg/kg) into the influenza A virus (A/FM/1/47)-infected mice. This study noticed the improved survival rate of the infected mice. The humoral immune response is detected by using an enzyme-linked immunosorbent assay (ELISA) which detects the elevation of IgA, IgM, and IgG antibodies. The flow cytometric analysis is used to detect cell-mediated immune response which results in the increased CD3+ and CD4+ T cell levels. An increase in the production of anti-inflammatory cytokines IL-10 and IFN-γ reveals the reduction of lung inflammation due to infection [122].

Wu et al. [123] investigate in vitro, in vivo, and in silico studies on Patchouli alcohol (PA) against influenza A virus (A/Leningrad/134/17/1957, H2N2). In vivo, MTT assay reveals PA at IC50 of 4.03 ± 0.23 μM inhibits the viral penetration. Through in vivo, PA at a dose of 5 mg/kg/day protects the infected mice, comparable with Oseltamivir. In silico docking and molecular dynamic simulations exhibit PA binding in the Asp151, Arg152, Glu119, Glu276, and Tyr406 active site regions of H2N2 NA with interaction energy of – 40.38 kcal mol^–1^.

#### Triterpene

The study of Hong et al. investigates triterpene compounds, Betulinic acid (BeA), of *Zizyphus jujuba* have antiviral activity against influenza A/PR/8–infected A549 cell line and C57BL/6 mice. In in vivo study, the SRB method assesses proliferation inhibition in a dose-dependent manner (0.4–50 μM) in A549 cells without major cytotoxicity. Intraperitoneally administering BeA (10 mg/kg/dose) into the infected mice reveals the attenuation of increased necrosis, inflammatory cells, and pulmonary edema. ELISA study marks the decreased level of inflammatory cytokine IFN-γ level in BeA-treated infected mouse lung [150].

#### Meroterpene

Bakuchiol is a meroterpene compound derived from *Psoralea carylifolia*. Shoji et al. investigate naturally occurring (+)-(S)-bakuchiol and synthetic enantiomer (−)-(R)-bakuchiol against influenza A viral strains A/PR/8/34, A/CA/7/09 (H1N1), and A/Aichi/2/68 (H3N2) and observed (+)-(S)-bakuchiol inhibited the H1N1 strains but not the H3N2 strain by inhibiting viral growth, decreased viral mRNAs and protein expression in MDCK cells. Naturally occurring (+)-(S)-bakuchiol shows greater anti-influenza activity than enantiomer (−)-(R)-bakuchiol. Bakuchiol induced Nrf2 activation and upregulated *NQO1*, p53, and *GSTA3* mRNA levels in MDCK cells thereby inhibiting viral growth [125].

#### Terpenoids

Terpenoids are a modified class of terpenes with various cyclizations and rearrangement of the carbon skeleton [165, 166]. Sesquiterpenoids, diterpenoids, and triterpenoids are the various types of terpenoids. Terpenoid-derived drugs gained significant attention towards the treatment of human infectious diseases [167].

#### Sesquiterpenoids

Sesquiterpenoids are derived from sesquiterpenes showing diverse hydrocarbon backbone [168].

The Sesquiterpenoid Germacrone is one of the active components found in the rhizome of *Curcuma longa*. Liao et al. explain the antiviral activity of Germacrone against influenza A viruses (A/PuertoRico/8/34, A/human/Hubei/1/2009, A/human/WSN/33 (H1N1), A/human/Hubei/3/2005 (H3N2), and influenza B viruses (B/human/Hubei/1/2007) in MDCK and A549 cells by the dose-dependent manner with EC_50_ of 6.03 μM. The neuraminidase assay in A549 cells and plaque reduction assay show reduced NA and plaque numbers. Through indirect immunofluorescence assay, reduction of viral protein expression, RNA synthesis, and the progeny viruses observed both in MDCK and A549 cells. In a time-of-addition study, the early stage of viral inhibitory activities was observed. Germacrone administered at 100 mg/kg provides effective protection. Moreover, the studies suggested Germacrone in combination with oseltamivir produces a significant effect [92].

#### Diterpenoid

Diterpenoid compounds belong to the Terpenoid class [169] that is produced by diterpene synthases (diTPSs) and other enzymes resulting in a wide structural diversity [170]. Generally, these compounds are odorless with strong flavors.

Andrographolide is a Diterpenoid obtained from *Andrographis paniculata.* Recent studies show Andrographolide derivatives have potent activity opposing influenza infection. 14-a-lipoyl andrographolide (AL-1) is the synthesized Andrographolide derivative studied by Chen et al. against influenza A viruses (A/Chicken/Guangdong/96 (H9N2), A/Duck/Guangdong/99 (H5N1), and A/PR/8/34 (H1N1)) in MDCK cells. The in vivo plaque reduction assay using EC_50_ of 7.2 to 15.2 mM exhibited the most effective antiviral activities in MDCK cells. Hemagglutination assays show a minimum inhibitory concentration range of 5.3–16.8 mM indicate that AL-1 directly inhibits hemagglutinin and prevents virus adsorption. In vivo render positive information like prolonged survival, decreased lung consolidation of infected mice by oral gavages of AL-1 ranged from 100 to 200 mg/kg/days. The best result was obtained by administered twice daily for 7 days beginning 24 h before viral exposure [72].

Another Andrographolide derivatives compound, 14-deoxy-11,12-dehydroandrographolide (DAP), has potency against influenza A viruses (A/chicken/Hubei/327/2004, A/duck/Hubei/XN/2007 (H5N1), A/PR/8/34, A/NanChang/08/2010 (H1N1), and A/HuNan/01/2014 (H3N2)) in vitro, with CC_50_ values of 77 and 243 lg/mL in both A549 and MDCK cells. CCK-8 assay detects DAP exerts no cytotoxic effect on the cells and the morphology of the cells remains unaffected. But it shows a pronounced inhibitory effect on viral progeny. Further analysis of DAP is continued with H5N1 virus (A/chicken/Hubei/327/2004) where viral nucleoprotein (NP) mRNA, NP, and NS1proteins were significantly inhibited but it does not show any effect on the absorption from A549 cells. Immunostaining assay explores Dap effective control in inhibiting nuclear export of viral ribonucleoprotein (vRNP) complexes. The qRT-PCR reduction of proinflammatory cytokines (TNF-a, IL-6, IL-8, IFN-a, IL-1b, and IFN-b) and chemokines (CXCL-10 and CCL-2) by DAP was observed [73].

#### Triterpenoids

Triterpenoids are the oxygen derivatives of triterpenes [171]. Triterpenoids 3*α*-hydroxylup-20(29)-ene-23,28-dioic acid and 3-*epi*-betulinic acid 3-*O*-sulfate isolated from *Schefflera heptaphylla* profoundly active against influenza A (H1N1) virus with IC_50_ values of 25 and 31.3 μg/mL [131].

#### Alkaloids

Alkaloids are nitrogen-containing secondary metabolites. Most of the alkaloids are pharmacologically active compounds [172]. Some of the noted alkaloids like dendrobine of *Dendrobium nobile*, Indirubin of *Isatis indigotica and Strobilanthes cusia*, Lycorine and Hemanthamine of *Lycoris radiate*, Quinalizidine alkaloids of *Sophora* species, ormosinine appraise for their efficacy against influenza viruses.

The plant *Dendrobium nobile* stem is enriched with alkaloids namely Dendrobine. Li et al. expose dendrobine anti-influenza activity against H1N1 (A/FM-1/1/47, A/Puerto Rico/8/34 H274Y) and H3N2 (A/Aichi/2/68) with IC_50_ values of 3.39 ± 0.32, 2.16 ± 0.91, 5.32 ± 1.68 μg/mL, respectively. dendrobine inhibited the production of viral HA mRNA, HA, and NP proteins in both MDCK and A549 cells in a dose-dependent manner. It inhibited the early stage of viral replication by binding with the viral NP (Affinity constant K_D_ value: 1.19 × 10−2 μg/mL) thereby suppress its export resulting in the deactivation of the vRNP complex. In support of these findings, a docking study performed using AutoDock software revealed the binding of dendrobine in the highly conserved region of viral NP [95].

Herbs like *Isatis indigotica and Strobilanthes cusia* are enriched with Indirubin pigment used to treat respiratory viral infection. Mak et al. displayed the anti-influenza virus activity by inhibition of RANTES in influenza A (A/NWS/33)-infected H292 cells. It interrupts virus-induced NF-κB translocation and p38 MAP kinase activation [106].

*Lycoris radiate* bulbs have been used as traditional Chinese medicine. He et al. isolated and identified 15 alkaloids from it using spectroscopic analysis. Among these, four alkaloids lycorine, hippeastrine, hemanthamine, and 11-hydroxyvittatine exhibited antiviral activities against H5N1 (A/Chicken/Guangdong/178/2004) in MDCK cells with EC_90_ values of 0.52, 82.07, 4.15, and 13.45 μm. From these Lycorine and Hemanthamine were further analyzed which block vRNP nuclear export [108].

In another study, Lycorine and Hemanthamine were further investigated by He et al. These compounds exert antiviral activity against H5N1 (A/CK/GD/178/04, A/DK/GD/212/04), H3N2 (A/Swine/GD/166/06), H1N1 (A/CK/HN/170/03, A/PuertoRico/8/34), H9N2 (A/CK/GD/400/07, A/CK/GD/228/04) in MDCK cells after the viral entry. These drugs display cytostatic effects by resisting pro-apoptotic stimuli. The virus-infected cell growth slowed down by arresting the cell cycle at the G1/S phase and its cytoskeleton arrangement changed. This showed the target of the viruses is the cytoskeleton in particular the monomer Actin. The compound-treated infected cells displayed a decrease in the S phase of the cell cycle and protect against cytoskeleton disruption [109].

*Sophora* species of Leguminosae family endowed with quinolizidine alkaloids exhibit anti-influenza activity. This is evident from the study of Dang et al. against H1N1 (A/Puerto Rico/8/34) virus and H3N2 Oseltamivir-sensitive virus (VR1679) which shows dihydroaloperine potent inhibitory activity with EC_50_ of 11.2 μM targeting NP. Interestingly, chemical modifications on the N12 and C16 positions of the aloperine scaffold improve the efficacy approximately about 5-fold [140].

On more quinolizidine alkaloids, Matrine of *Sophora flavescens* has been proposed to be a target for the development of anti-influenza drugs. Yang et al. made the first attempt to utilize the molecular imprinted polymer technique, a non-biological method to identify the active components against the influenza virus. In this study, chloroform extracts of chlorogenic acid, phillyrin, matrine, oxymatrine, sophoridine, oxysophoridine, and aspirin were used as test compounds. Synthesized Oseltamivir molecularly imprinted polymer (OSMIP) was applied to liquid chromatography (LC) which exposed the retention time of 140 min used as template (OSMIP-LC column) for the selection of test compounds. The compound polarity was detected by OSMIP-LC column online and by electrospray ionization (ESI) MS offline shows Matrine with m/z 249 shows similar correlation like Oseltamivir. Further, in vitro study revealed matrine efficacy against H9N2 (A/Goose/Dalian/3/2001) in reducing cytotoxic effect and HI inhibition similar to Oseltamivir. Stereostructural resemblance consent matrine with Oseltamivir [141].

In silico studies of Gangopadhyay et al. [151] explain the alkaloid ormosinine might be a potential inhibitor of wild-type and mutant haemagglutinins of H5N1 virus.

#### Phenolic compounds

Phenolic compounds are the major secondary metabolite classified into several types as Phenylpropanoid, Tannin, Catechol, and Anthraquinone [173].

#### Phenylpropanoid

Phenylpropanoids comprise a wide diverse group of secondary metabolites which are synthesized from the primary metabolites, phenylalanine or tyrosine amino acids, either through the shikimate or the phenylpropanoid pathway. Phenylpropanoids can be categorized into five different groups as Flavonoids, Lignans, Phenolic acids, Stilbenes, and Coumarins [174].

#### Flavonoid

Flavonoids are the major group of Phenylpropanoids which comprise 8000 and more compounds [175]. The basic skeleton structure of flavonoids is C6-C3-C6 having a fused benzene ring designated as ring A and the pyran as ring c, along with a phenyl group as ring B [176]. Flavonoids classified into three different classes as Flavonoids have 2-phenylbenzopyrans, Isoflavonoids with 3-benzopyrans, and neoflavonoids with 4-benzopyrans [177]. Besides these, Biflavonoid [178] and prenylflavonoid [179] are the other classes included under flavonoid.

#### Flavonoid (2-phenylbenzopyrans)

Flavonoids are the natural compounds widely found in fruits, vegetables, flowers, seeds, nuts, spices, stems, chocolate, tea, and wine [180]. They have become an indispensable component in drug discovery research and have been ascribed promising resources for combating several viral diseases including influenza [181]. They are subdivided into different subgroups as flavones, flavanones, flavonols, flavanonol, flavan-3-ol, anthocyanin, and chalcones [182].

#### Flavones

Flavones are widely distributed in plants in various forms. Medicinal plants like *Scutellaria baicalensis* are reported to have the flavones Baicalin, 5, 7, 4′-trihydroxy-8-methoxyflavone (F36), isoscutellarein (5,7,8,4′-tetrahydroxyflavone), wogonin, apigenin, baicalein, and chrysin shows anti-influenza activity. Apigenin and luteolin of *Elsholtzia rugulosa*, luteolin of *Perilla frutescens*, quercetin 3-rhamnoside (Q3R) of *Houttuynia cordata*, newly identified flavones of *Sambucus nigra*, and myricetin of *Aronia melanocarpa* are noted for their anti-influenza activities.

The herb *Scutellaria baicalensis* is enriched with flavones like baicalin. Ding et al. proved the efficacy of baicalin against influenza H1N1 (A/FM1/1/47) and H3N2 (A/Beijing/32/92) viruses by both in vitro and in vivo studies. The in vitro study provide EC_50_ values 43.3 lg/mL and 104.9 lg/mL and the NI assay shows IC_50_ values 52.3 lg/mL and 85.8 lg/mL for the H1N1 and H3N2 viruses, respectively. These results explain baicalin as an effective neuraminidase inhibitor. Baicalin affected the virus budding in MDCK cells inoculated with the virus. Intravenous baicalin injection proved an increased survival rate with improved lung parameters in influenza virus–infected mice [132].

Neuraminidase enzyme also called sialidase cleaves the sialic acid link, between the virions and the host cell surface thereby maintaining the cycle of infection [183]. Nagai et al. checked the Sialidase inhibitory activity of flavonoids about 103 species using sodium p-nitrophenyl-N-acetyl-z-d-neuraminate as substrate and found 5, 7, 4′-trihydroxy-8-methoxyflavone (F36) from the roots of *Scutellaria baicalensis* as potent inhibitor (IC_50_ μM). Besides that, it blocked the infection and replication of the A/PR/8/34 influenza virus [133].

The authors showed the sialidase inhibitory effect of isoscutellarein (5,7,8,4′-tetrahydroxyflavone) isolated from the leaf of *Scutellaria baicalensis* (Ki 41 μM) and observed replication inhibition in A/WSN/33 and A/PR/ 8/34 viral strains in MDBK cells (IC_50_ 20 pM) and allantoic sac of an embryonated egg. The authors observed Isoscutellarein produce a similar effect as F36 and administered both into the mouse-adapted A/PR/8/34. Intranasal administration disclosed F36 (0.5 mg/kg) complete prevention of virus proliferation, intraperitoneal administration showed F36 (4 mg/kg) reduced lung virus titers to 10^<2^, and oral administration explicit isoscutellarein (400 and 40 mg/kg) significantly reduced lung virus titers [134].

The author continues further study by isolating 5, 7, 4′-trihydroxy-8-methoxyflavone (F36) from the roots of *Scutellaria baicalensis* and noted its effect in the reduction of H3N2 (A/Guizhou/54/89) and B (B/Ibaraki/2/85) viruses in MDCK after 4 hours of incubation. It reduces the virus yield by 50% at 20 μm and 90% at 40 μm in a dose-dependent manner. Fusion assay reported F36 was the inhibitor of virus fusion with the endosome/lysosome of the host cell. Intranasal 7 times (3.5 mg/kg) administration of F36 completely prevented the proliferation but did not inhibit proliferation of mouse-adapted with virus inoculation from 18h before to 54 h after [135]. The investigators perform the same mode of research work by applying F36 (50μm) on mouse-adapted influenza virus A/PR/8/34 (A/PR8) and obtain a similar result like progeny inhibition and endosome/lysosome membrane fusion inhibition [136].

Seong et al. investigated the antiviral role of wogonin isolated from *Scutellaria baicalensis* against influenza H1N1 (A/Puerto-Rico/8/34); seasonal H1N1 and H3N2; and B of Yamagata lineage (clinical influenza strains) and observed replication suppression in virus-adapted MDCK and A549 cell lines. Wogonin (10 μg/mL)-treated influenza A virus–infected cells had significantly increased transcript levels of IFN-β and IFN-λ along with IFN downstream molecules MxA (myxovirus resistance gene A) and OAS (2-5′ oligoadenylate synthetase). Furthermore, wogonin inhibited influenza virus–induced AMPK (5′ adenosine monophosphate–activated protein kinase) phosphorylation [137].

Hour et al. carried out in vitro and in silico studies using MeOH, EtOAc, and chloroform extracts of *Scutellaria baicalensis* against pandemic 2009 H1N1 (A/Taiwan/CMUH/2009), seasonal H1N1 (A/Taiwan/CMUH/2007, seasonal strains), seasonal H3N2 (Seasonal strains), and H1N1 (influenza A/Puerto Rico/8/34). EtOAc (IC_50_23.7 to 27.4 μg/mL). The chloroform extracts (IC_50_ 14.16 to 41.49 μg/mL) considerably decreased the virus proliferation than MeOH extract. The EtOAc (IC_50_73.16 to 487.40 μg/mL) extract was remarkably noted for NA inhibition. HPLC analysis reported the flavone baicalin highly prevailed in the MeOH extract, high presence of apigenin in EtOAc, whereas in the EtOAc, and chloroform extracts were enriched with the flavones baicalein and chrysin. Further, in vitro and in silico studies are carried by using the flavones baicalin, apigenin, baicalein, and chrysin. In silico molecular simulation studies of these flavones against NA reveal baicalein potent interaction with NA1 active sites. In vitro plaque reduction assays also suggest baicalein effectively inhibit replication of pandemic 2009 H1N1 (IC_50_ = 0.02 μM) and seasonal 2007 H1N1 (IC_50_ = 0.018 μM) [138].

Liu et al. isolated five flavonoids, namely apigenin (1), luteolin (2), apiin (3), galuteolin (4), and luteolin-3′-glucuronyl acid methyl ester (5) from the EtOAc extract of *Elsholtzia rugulosa*. The maximal non-cytotoxic concentration (MNCC) of the EtOAc extract with IC_50_ 55.56 μg/mL and its constituents showed inhibition of H1N1 (A/PR/8/34), H3N2 (A/Jinan/15/90), and B (B/Jiangsu/10/2003). Among them, apigenin (IC_50_ 1.43 μg/mL) and luteolin (IC_50_ 2.06 μg/mL) exhibited the highest activities against H3N2 [96].

*Perilla frutescens* seeds contain four main polyphenolic compounds namely, rosmarinic acid-3-*O*-glucoside, rosmarinic acid, luteolin, and apigenin which were tested for the antiviral potential against recombinant virus H1N1 neuraminidase (rvH1N1 NA E.C. 3.2.1.18). Of these, the flavones luteolin (IC_50_ 8.4 μM) and the rosmarinic acid (Phenolic acid with IC_50_ of 46.7 μM) showed potent NA activity and proved as noncompetitive inhibitors [118].

Choi et al. investigated the antiviral effects of the flavone quercetin 3-rhamnoside (Q3R) from *Houttuynia cordata* against H1N1 strain A/WS/33 and observed the reduction of cytopathic effect in virus-infected MDCK cells. Pre-exposure of virus or Pre-inoculation of MDCK cells with Q3R did not alter its infectivity but Q3R role is appreciable only added at 1, 2, and 4 h after virus inoculation with the reduction in viral mRNA synthesis. This made the investigators conclude Q3R inhibits the initial stage of viral replication [104].

In vitro studies of Roschek B Jr et al. reveals the inhibitory efficiency of *Sambucus nigra* extract (IC_50_ 252 ± 34 μg/mL) against H1N1(A/PR/8/34) and identified two flavonoid compounds 5,7,3′,4′-tetra-O-methylquercetin (1) and 5,7-dihydroxy-4-oxo-2-(3,4,5-trihydroxyphenyl) chroman-3-yl-3,4,5-trihydroxycyclohexanecarboxylate (2) through direct binding assay and DART TOF-MS analysis. The authors urged these compounds were primarily responsible for anti-influenza activity by synthesizing flavanonol compound 1 and a flavanonol compound dihydromyricetin (corresponding 3-hydroxyflavonone of 2) and observed H1N1 inhibition with IC_50_ value of 0.13μg/mL and 2.8μg/mL. These compounds directly bind to the virus particles and prevent host cell entry and/or recognition similar to the antiviral drugs Oseltamivir and Amantadine [130].

*Aronia melanocarpa* fruits are rich in polyphenolic compounds which displayed wide antiviral activity against H1N1, H3N2, recombinant H1/PR8 expressing green fluorescent protein (rPR8-GFP), and influenza B virus. The *Aronia* mode of virucidal action is targeting the HA protein of H1N1 (A/Korea/01/2009) which is evident from the HI assay. Its flavone myricetin and the phenolic acid compound ellagic acid successfully inhibited the replication and did not show any cytotoxic effect against MDCK cells. The in vivo study exhibited myricetin and ellagic acid increase the survival rate by 50% and 37.5% of rPR8-GFP virus–infected mice [75].

#### Flavonols

Flavonols exist as Hyperoside in *Azadirachta indica*, Gossypetin and Kaempferol in *Rhodiola rosea*, and quercetin of *Chaenomeles speciosa* endowed as a promising agent for suppressing viral activities.

Ahmad et al. perform in silico docking studies by selecting *Azadirachta indica* active compounds nimbaflavone, rutin, and hyperoside along with drugs OMS, CBX, LGH, naproxen, BMS-883559, and BMS-885838 as ligands and the nucleoprotein structure [PDB ID:3RO5] as a receptor. All these ligands displayed perfect binding by interacting in the conserved residue (ASP302, TYR52, SER50, GLY288, SER376, and ARG99) of the receptor. But the best interaction was obtained from hyperosides along with the drugs LGH, naproxen, BMS-885838, and BMS-883559 [77].

*Rhodiola rosea* is a herbaceous plant used for food and medicine which contains constituents like flavonoids, phenylpropanoid and phenyl ethanol derivatives, and aliphatic glycosides. In this study, Jeong et al. isolated five flavonols (3, 5, and 9–11), and their structure-activity relationship (SAR) is compared with available flavonoids (1, 2, 4, 6–8, and 12–14). All these compounds unveiled NI activity against *Clostridium perfringens* (IC_50_ 0.8 to56.9 μM) and rvH1N1 (IC_50_ 2.2 to 56.5 μM). The antiviral activities of the compounds 1–6, 8–12, and14 had EC_50_ values of 30.2–99.1 μM against H1N1 (A/PR/8/34) and 18.5–103.1 μM against H9N2 (A/Chicken/Korea/MS96/96) in MDCK cells. Of these compounds, gossypetin (6) exhibited the most potent NI inhibitory activity (IC_50_ values of 0.8 and 2.6 μM) and kaempferol (3) exhibited the highest anti-influenza activity against H1N1 and H9N2 (EC_50_ values of 30.2 and 18.5 μM). The kinetic studies exposed all isolated flavonols act as non-competitive inhibitors [127].

The flavonol quercetin of *Chaenomeles speciosa* exhibited significant dose-dependent DPPH radical scavenging activity with IC_50_ 3.82 μg/mL. Quercetin of 5μg/mL inhibits TNF-α by 33.14% and NO production [88].

#### Flavanonols

Flavanonol dihydromyricetin isolated from *Sambucus nigra* activated and induced the immune system to combat the influenza virus H1N1 (A/PR/8/34) through binding with virions and blocking entry and recognition of the host cell [130].

#### Flavan-3-ol

Flavan-3-ol compounds catechin, epicatechin (EC), epigallocatechin (EGC), epicatechin-3-gallate (ECG), epigallocatechin-3-gallate (EGCG), (−) epigallocatechin gallate (EGCg), and theaflavin digallate (TF3) have been reported in *Camellia sinensis*, (+)-catechin of *Ephedrae herba*, catechin-3-O-gallate of *Polygonum cuspidatum*, and gallocatechin-7-gallate (J10688) of *Pithecellobium clypearia* contribute significantly against influenza viruses.

Tea is produced from the leaves of *Camellia sinensis* and classified into three types as non-fermented green tea, semi-fermented oolong tea, and fermented black and red tea [184]. The green tea is enriched with flavonol catechin. There are four main types of catechin predominantly found in green tea, namely epicatechin (EC), epigallocatechin (EGC), epicatechin-3-gallate (ECG), and epigallocatechin-3-gallate (EGCG) [185]. Differing from that, the black tea contains the polymerized catechins, namely theaflavins and thearugins [184]. Worldwide Green tea consumption is increasing due to the awareness of its health benefits [186]. Park et al. conducted a population-based study of school children in Kikugawa City, Japan, using the anonymous questionnaire survey and concluded the work that consumption of 1–5 cups/day of green tea might prevent influenza infection among schoolchildren in a tea plantation area [187]. Matsumoto et al.’s studies explored green tea catechin prevents influenza among 197 health care workers [81].

Imanishi et al. studied the additional inhibitory effect of green tea extract (GTE) in MDCK cells infected with H1N1 (A/PR/8/34), H3N2 (A/Aichi/2/68), and B (B/Singapore/222 (Sing) viruses and observed acidification of ELS (endosome and lysosome) inhibition through vital fluorescence microscopic studies. GTE exerts its effect within 5–15 min after infection and the authors observed the same inhibitory effects in GTE active catechin component (-) epigallocatechin (EGC) [82].

Green tea catechin compounds (−)-epigallocatechin gallate (EGCG), (−)-epicatechin gallate (ECG), and (−)-epigallocatechin (EGC) were analyzed for their anti-influenza activities against HIN1 (Influenza A/Chile/1/83, H3N2 (A/Sydney/5/97) and B (B/Yamagata/16/88) viruses. EGCG (EC_50_22–28 μM) and ECG (EC_50_22–40 μM) showed more potent inhibition against the viruses than EGC (EC_50_ 309–318 μM), and also, more effective HI and NI activity and suppression of viral RNA synthesis in MDCK cells were noted in EGCG and ECG than in EGC [83].

The catechin (–)-epigallocatechin-3-O-gallate(EGCG) and EGCG-C-16 showed broad-spectrum anti-influenza activity against H1N1 (A/PuertoRico/8/34, A/Beijing/262/95, Yokohama/77/2008, Yokohama/63/2007, A/Yokohama/91/2008), H3N2 (A/Panama/2007/99), H5N2 (A/Duck/HongKong/342/78), and B (B/Yamanashi/166/98/) viruses. EGCG-C-16 EC_50_ values range between 10 and 61 nM which were 7.1- to 44-fold lower than EGCG and CC_50_ value was 82 μM which was 3.1-fold lower than EGCG. Hence, EGCG-16 proved as a potent inhibitor with an SI value 2.2- to 14-fold greater than EGCG [84].

Nakayama et al. purified (−) epigallocatechin gallate (EGCg) and theaflavin digallate (TF3) from green and black tea and performed an inhibitory activity in opposition to H1N1 (A/Yamagata/120/86) and B (B/USSR/100/83) virus–infected MDCK cells. Virus agglutination was observed when it was mixed with EGCg and TF3 even at a low concentration of 1.5 μM and prevents its adsorption to MDCK cells. EGCg and TF3 at the concentration of 1–16 μM bind to HA, thereby preventing haemagglutination and obstructing its infectivity. However, EGCg (> 200 μM) and TF3 (> 100 μM) were toxic to MDCK cells [85]. In support of the above findings, Sahoo et al. perform in silico docking studies and concluded theaflavin as a potent natural inhibitor bind with the lowest energy (–5.21 kcal/mol) to H1N1 neuraminidase [86].

Mantani et al. observation reveal (+)-catechin(1.0-10.0 mM) in the extract of *Ephedra* herba(EHext) inhibits acidification of ELS of H1N1(APR/8/34) at 1 h. Effective inhibition of virus growth was observed when (+)-catechin (1.25–10.0 mM) treated within 10 min p.i. Surprisingly, 1h or later, treatment exerted little and reversible inhibitory effect [97].

Chen et al. isolated and identified seven compounds 2-methoxystypandrone (1), emodin (2), resveratrol(3), polydatin (4), emodin-8-O-beta-d-glucopyranoside (5), (E)-3, 5, 12-trihydroxystilbene-3-O-beta-d-glucopyranoside-2′-(3″, 4″, 5″-trihydroxybenzoate) (6), and catechin-3-O-gallate (7) from the ethyl extract of *Polygonum cuspidatum*. These compounds were treated with H1N1-affected MDCK cells and revealed compounds 6 (EC_50_ 5.9μM) and 7 (EC_50_ 0.9μM) as potent inhibitors exhibiting low cytotoxicity to the host cells. The NI assay identified compounds 3, 6, and 7 with IC_50_ values of 129.8, 44.8, and 21.3 as active NA inhibitors [124].

*Pithecellobium clypearia* is rich in flavonoid compounds. Kang et al. isolated and identified the flavan-3-ol compounds (2R,3R)-7-O-galloylplumbocatechin A (1), three known flavonoids (−)-5,3′,4′,5′-tetrahydroxyflavan-7-gallate (2), (+)-3,5,3′,4′,5′-penta-hydroxyflavan-7-gallate (3), and (−)-7,4′-di-O-galloyltricetiflavan (4) from the leaves and twigs. These compounds were examined for NA assay, against H1N1 (A/PR/8/34), H3N2 (A/Sydney/5/97), and B (B/Jiangsu/10/2003) viruses. The result indicated compounds 1 (IC_50_ 29.77 ± 6.12 μg/mL) and 2 (IC_50_ 36.91 ± 3.80 μg/mL) exhibited moderate activity against H1N1. All the compounds inhibited the expression of proinflammatory cytokines IL-6 or MCP-1 induced in H1N1 virus–adapted A549 cells. The structural verification of the compounds was performed using spectroscopic analysis [119].

From the leaves and twigs of *Pithecellobium clypearia*, Li et al. isolated flavan-3-ol gallocatechin-7-gallate (J10688) which acts as a novel CLK1 inhibitor with potent anti-influenza activity. In vivo studies performed by using intravenous administration of J10688 (30 mg/kg/day) in H1N1-infected ICR mice which showed inhibition of pro-inflammatory cytokines IFN-γ, IL-6, TNF-α, and IL-1β, thereby preventing the viral infection and lung injury, and increased survival rate (91.67%). In addition to this, dose-dependent treatment of J10688 stimulated lymphocyte proliferation, thereby cellular and humoral immune responsees were improved. In vivo cytotoxic studies performed by using J10688 against the strains H1N1 (A/PR/8/34), H3N2 (A/Sydney/5/97), and B (B/Jiangsu/10/2003) showed EC_50_ values 1.69, 2.28, and 23.18 μM, respectively. Hence, further anti-influenza studies were conducted by using H1N1-infected A549 cells where J10688 dose-dependently (3, 10, 30, μmol/L) decrease viral NP, M2, and RNA synthesis. Alternative splicing of the influenza virus M2 gene depends on the host cdc2-like kinase 1 (CLK1). J10688 was an effective CLK1 inhibitor, by downregulating SC35 and SF2/ASF excessive phosphorylation and thereby controlling M2 mRNA alternative splicing [120].

#### Anthocyanin

Anthocyanin compounds from *Ribes nigrum* and red-fleshed potato impart inhibitory activity against influenza viruses.

A study performed by Li et al. showed that using *Ribes nigrum* crude extracts with a pH 7.2 inhibit H1N1 (A/PR/8/34) and B (B/Gifu/2/73) plaque formation by 50% of IC_50_ 3.2μg/mL and directly inactivated 99% of the virus by 10μg/mL with a pH 2.8. Post-exposure treatment of the extracts presented complete inhibition suppression in H1N1 (10–100μg/mL) which indicates the extract suppresses the late stage of virus growth. After 8–9 h of infection (both IVA and IVB) showing MDCK cells are treated with the extract of 100 μg/mL for 1 h, and the virus titers in culture fluids show complete inhibition indicates extract inhibits the virus release [128].

The same authors and other investigators further investigated the crude extract and found anthocyanins as major constituents responsible for anti-influenza activity. Using TLC, the extract was fractionated into A to D and Anthocyanin D fraction was further fractionated into A′ to G′. The fractions D′ to G′ produced potent anti-influenza activity against A and B viruses. E′ and F′ exposed additive antiviral effects and identified as 3-O-alpha-L-rhamnopyranosyl-beta-d-glucopyranosyl-cyanidin and 3-O-beta-d-glucopyranosyl-cyanidin, and 3-O-alpha-L-rhamnopyranosyl-beta-d-glucopyranosyl-delphinidin and 3-O-beta-d-glucopyranosyl-delphinidin using HPLC. F′ inhibits virus adsorption, release but not directly inactivate it [129].

Hayashi et al. bred anthocyanin-rich tetraploid potatoes cultivars of *Solanum tuberosum ssp. tuberosum* and *S. tuberosum ssp. Andigena* and red-fleshed potato (Inca Red) were selected. The purified anthocyanin powder along with the pigments pelanin (3-*O*-[6-*O*-(4-*O*-p-coumaroyl-α-L-rhamnopyrano-syl)-β-d-glucopyranosyl]-5-*O*-β-d-glucopyranosyl-pelargoni-din), pelargonidin, pelargonidin 3-p-coumaroylglucose with 5-glucose, and pelargonidin 3-p-coumaroylglucose with 5-malonylglucose were tested for anti-influenza activity against H1N1(A/PR/8/34) and B(B/Gifu/2/73). The result revealed that anthocyanin powder showed the best inhibitory activity against H1N1 (IC_50_ 48μg/mL) and B (IC_50_ 54 μg/mL) than other pigments. The authors suggested that the antiviral activity of the potato anthocyanin is due to its additive or synergistic effect on other constituents including anthocyanin [139].

#### Chalcones

Chalcones bestowed with therapeutical properties have been noted for their anti-influenza activities. Recently chalcones isolated from *Glycyrrhiza uralensis*, *Glycyrrhiza inflata*, *Thalassodendrin ciliatum*, and *Caesalpinia sappan* showed strong anti-influenza activities.

Ryu et al. isolated 18 polyphenol compounds consisted of four chalcones, nine flavonoids, four coumarins and one phenylbenzofuran from the roots of *Glycyrrhiza uralensis* and evaluate their NA inhibitory activity. Of these the chalcone isoliquiritigenin (IC_50_ 9.0μM) and coumarin glycyrol (IC_50_ 3.1μM) showed strong inhibition on rvH1N1 NA (A/Ber-vig_Mission/1/18) and these are proved to be non competitive inhibitors [100].

From the acetone extract of *Glycyrrhiza inflata*, 8 chalcones were isolated, and their NA (H1N1, H9N2, H1N1 (WT), and H1N1 (H274Y)) inhibitory activities were accessed. Of these, compounds 3 (echinantin) and 6 (isoliquiritigenin) without the prenyl group exhibit strong inhibitory activity in viruses-infected 293T cells. Compound 3 (echinantin) and oseltamivir proved synergistic effects against NA of H274Y virus [99].

From the EtOAc extract of *Thalassodendron ciliatum*, Thalassodendrone (dihydrochalcone diglycoside), and 5 known phenolic compounds, namely asebotin, quercetin 3,7 diglucoside, protocatechuic acid, ferulic acid, and p-hydroxybenzoic acid were isolated and identified. These compounds were evaluated for anti-influenza activity against H1N1 (influenza A virus /WSN/33) and revealed asebotin (IC_50_ 2.00μg/mL, CC_50_ 3.36 μg/mL) inhibition and cytotoxic concentration were more than those of thalassodendrone (IC_50_ 1.96 μg/mL, CC_50_ 3.14 μg/mL) [147].

Liu et al. isolated six NA inhibitor compounds that are brazilein, brazilin, protosappanin A, 3-deoxysappanchalcone, sappanchalcone, and rhamnetin from *Caesalpinia sappan* and evaluated their anti-influenza activity against A/PR/8/34 (H1N1), A/Guangdong/243/72 (H3N2), and B/Jiangsu/10/2003 viruses. The results disclosed that the chalcone compounds 3-deoxysappanchalcone (IC_50_ 1.06) and sappanchalcone (IC_50_ 2.06 μg/mL) explained the highest inhibition activity against H3N2-infected MDCK cells [79].

#### Isoflavonoid (3-benzopyrans)

Isoflavonoid compounds build great attention in health-protecting and health-promoting effects preferable to use in human medicine [188]. This class includes pterocarpans [189] and homoisoflavonoids [190].

#### Pterocarpans

From the methanol extract of *Sophora flavescens*, three pterocarpans and six flavanones were isolated and their NA inhibitory activity disclosed all the isolated compounds excluded the compound 3, showing inhibitory effects between 12 and 20 μM. Among these, pterocarpan 1 with IC_50_ 1.4 μM displayed the best inhibition activity. Moreover, molecular docking studies of pterocarpan 1 with the receptor neuraminidase (PDB 1L7F) show the binding region nearer to the active site of the receptor [142].

#### Homoisoflavonoid

Homoisoflavonoids are a rare class of flavonoids present in few plant families like Fabaceae, Asparagaceae, Portulacaceae, Cucurbitaceae, and Polygonaceae [191]. Recently, it is reported in *Caesalpinia sappan* of the Fabaceae family disclosing potential anti-influenza activities.

Jeong et al. isolated 12 homoisoflavonoid compounds from the dried heartwood of *Caesalpinia sappan* and their antiviral efficacy against H1N1 (A/PR/8/34), H3N2 (A/Hong Kong/8/68), and H9N2 (A/Chicken/Korea/MS96/96) describe the compounds are reversible non-competitive inhibitors. Homoisoflavonoids with an unsaturated group like sappanone A (2) and brazilin (12) unveiled higher NA activity than saturated sappanone B (3). Hence, the investigators conclude that the α, β-unsaturated carbonyl group in the A-ring is the main factor needed for NA inhibition [80].

#### Biflavonoid

Biflavonoids like Ginkgetin obtained from *Ginkgo biloba* inhibit the influenza virus sialidase. Miki et al. analyze the anti-influenza sialidase activity of ginkgetin, synthesized ginkgetin-sialic acid conjugates (6R, 6S, 7R, 7S, 8R, 8S, 9R, 9S), and 5, 7, 4′-trihydroxy-8-methoxyflavone (F36) against H1N1 (A/PR/8/34), H3N2 (A/Guizhou/54/89) and B (B/Ibaraki/2/85) viruses. Ginkgetin inhibited sialidase of H1N1 and H3N2 with IC_50_ values of 55.00 and 9.78 μg/mL whereas the conjugate 8R (IC _50_ 5.50 lg/mL and 0.82 lg/mL) showed considerable inhibitory power. Ginkgetin acts as a sialidase inhibitor but it exerts a cytotoxic effect in MDCK cells, while the conjugates 8R and 8S are empowered with low cytotoxic effect and high sialidase inhibition. The intranasal application of 8R and 8S to the H1N1-infected mice reflected an increased survival rate of 75–78% at day 10 and 62.5–56% at day 21 [98].

#### Prenylflavonoid

In the study by Chiou et al., prenylflavonoid 8-prenylkaempferol (8-PK) was isolated from the roots of *Sophora flavescens* and added into MDCK cells infected with H1N1 (A/PR/8/34) which significantly reduced RANTES accumulation along with nuclear factor-κB (NF-κB) and interferon regulatory factor 3 (IRF-3) nuclear translocation in a dose-dependent manner. The investigators revealed the fact that both NF-κB and IRF-3 were essential for H1N1 RANTES production. The influenza virus–activated PI3K pathway and Akt phosphorylation were also attenuated along with the prevention of IκB degradation by 8-PK. This PI3K-Akt pathway was essential for NF-κB- and IRF-3-mediated RANTES production [143].

#### Prenylflavanones

Ma et al. unveil that the six prenylflavanones, namely sophoraflavanones M(1) and N (2) glabranin (3) (2S)-7-hydroxy-5-methoxy-8-prenylflavanone(4), (2S)-8-[2-(3-hydroxyisopropyl)-5-methyl-4-hexenyl]-2′-methoxy-5,7,4′-trihydroxyflavanone (5), and leachianone B (6) from the roots of *Sophora flavescens* showed inhibition against H1N1 (A/WSN/33/2009) except compound 5 (EC_50_ 43.9 μM) which produced moderate inhibitory activity [144].

#### Lignans

Lignans are the polymers of lignin comprising a large number of compounds. Compounds include arctiin, arctigenin in *Arctium lappa*, clemastanin B, and lariciresinol-4-O-β-d-glucopyranoside from *Isatis indigotica* which played a predominant role to restrict influenza viral infections.

Hayashi et al. explored two lignan components, arctiin and arctigenin, isolated from the fruits of *Arctium lappa* which exerted strong inhibitory effects on the replication of the H1N1 (A/NWS/33) virus. Further ongoing investigation reveals that arctigenin exhibited a 6- to 8-fold lower IC_50_ value (IC_50_ 3.8–2.9 μM) with minimum cytotoxic effect (CC_50_ 45 μM) than arctin (IC_50_ 24–22 μM, CC_50_ 290 μM) in virus-infected MDCK cells. Thus, it will be interesting to investigate and characterize arctigenin through the time of addition assay and progeny release assay. Surprisingly, arctigenin’s best inhibitory effect was noted within immediate addition of virus-infected MDCK cells suggesting that it interacted with the early stage of viral replication but did not inhibit its cellular penetration. The addition of arctigenin, even after 8 h p.i. gives a scope that it inhibits viral progeny and release. The investigator’s in vivo study explicitly explains that the oral administration of arctiin was converted into arctigenin in virus-infected mice along with more production of antibodies against the influenza virus. The authors observed the synergistic effect of arctiin and oseltamivir decreased the virus yield in both bronchoalveolar lavage fluids and lungs of infected mice [74].

Yang et al. isolated the lignan clemastanin from the root of *Isatis indigotica* and evaluated its antiviral inhibition efficiency against influenza viruses, respiratory syncytial virus (RSV), adenovirus 3 (ADV3), parainfluenza virus 3 (PIV3), enterovirus 71 (EV71), and human rhinovirus (HRV). The authors observed the compound inefficiency against all the viruses except influenza viruses (H1N1, H3N2, H6N2, H7N3, H9N2, and influenza B) with IC_50_ 0.087–0.72 mg/mL. A distinct reduction of virus titer was observed only when the compound was added after viral incubation in the MDCK cell. The time course assay was performed using H1N1 (A/PR/8/34) and prominent inhibition was observed when clemastanin B treated 0–2 h after virus adsorption in the cell line, which suggested the compound target was at the early stage of replication. Moreover, this cell line unveiled the presence of viral RNP in their nucleus, which implies clemastanin B interferes with RNP export. Through a multi-passage experiment, the authors identified clemastanin B did not create drug resistance by the virus [107].

#### Phenolic acid

Phenolic acids are derivatives of benzoic acid and cinnamic acids. Benzoic acid derivatives include *p-*hydroxybenzoic acid, gallic acid, and ellagic acid, whereas cinnamic acid derivatives include rosmarinic acid [192].

The benzoic acid derivative compound 3, 4-dihydroxybenzoic acid of *Chaenomeles speciosa* shows significant dose-dependent DPPH radical scavenging and NA inhibition activities against H1N1. It also significantly inhibits *TNF-α* and NO production [88].

The water-soluble polyphenol tannin is found in various plants and brings antiviral activity. Theisen et al. demonstrate anti-influenza activity of ethanol extract of *Hamamelis virginiana* bark against various strains of influenza A virus (H1N1 A/Puerto Rico/8/34, pandemic H1N1 A/Luxembourg/46/2009, seasonal H3N2 A/Luxembourg/01/2005, H7N9 A/Anhui/01/2013) and found that gallic acid (phenolic acid), hamamelitannin (gallic acid derivative), tannic acid, and pentagalloylglucose (tannin) are the active part. The bark extract fractionated by ultrafiltration (UF) segregates low- and high-molecular-weight compounds. The low-molecular-weight compounds of less than 500 g/mol are gallic acid, epigallocatechin gallate, or hamamelitannin which exhibit neuraminidase inhibition but not hemagglutination, whereas high-molecular-weight tannin-containing extracts and tannic acid (1702 g/mol) show inhibition of viral binding and neuraminidase [101].

Ellagic acid of *Aronia melanocarpa* inhibits replication without cytotoxic effect in HIN1 (A/Korea/01/2009)-adapted MDCK cells and increased the 37.5% survival rate of rPR8-GFP virus-infected mice [75].

*Perilla frutescens* seeds contain rosmarinic acid, a cinnamic acid derivative, which shows potent NA activity with IC_50_ of 46.7 μM and proved as noncompetitive inhibitors [118].

#### Stilbenes

Resveratrol, a low-molecular-weight stilbene compound isolated from the ethyl extract of *Polygonum cuspidatum* act as NA inhibitors with IC_50_ values of 129.8 [124].

#### Coumarins

Kurokawa et al. selected six antipyretic compounds, 7-hydroxycoumarin (coumarin), 4-allylanisole, cinnamic acid ethylester, acetic acid cinnamylester, 2X-hydroxyacetophenone, and 2-hydroxycinnamic acid (cinnamyl derivatives) from the organic solvent-extractable fractions of *Cinnamomum cassia* and examined their antipyretic effects in H1N1 (A/PR/8/34)-infected mice that showed a remarkable reduction in rectal temperature. Further, four compounds, 7-hydroxycoumarin, 4-allylanisole, cinnamic acid ethylester, and acetic acid cinnamylester, suppressed the rise of interleukin-1α production to the basal level and reduced its circulation in the serum. The presence of the ester bond is the main determiner for both antipyretic and interleukin-1α suppression [90].

The above study brings out the fact that the coumarin compound, 7-hydroxycoumarin (7HC), reduced the pro-inflammatory cytokines along with antipyretic and interleukin suppression activities. The author Kurokawa and other investigators investigate 7HC in detail and explore 30 mg/kg oral administration of 7HC significantly reduces weight loss and virus production in the lung bronchoalveolar lavage fluid (BALF) of influenza-adapted mice. The author identified the suppression of pro-inflammatory and Th1 cytokine (IL-12 and interferon-gamma) results in the reduction of virus titer [91].

The coumarin glycyrol of *Glycyrrhiza uralensis* with IC_50_ 3.1 μM showed strong inhibition on rvH1N1 NA and proved to be a non-competitive inhibitor [100].

#### Tannin

The hydrolyzable tannins strictinin found in *Camellia sinensis* comprised about 0–1.0% dry weight. The study of Saha et al. revealed that strictinin inhibits influenza A and B virus replication in a dose-dependent way by using MDCK cells. The influenza strains used for the study are A/PuertoRico/8/34 (H1N1), A/Memphis/1/71 (H3N2), A/Aichi/2/68(H3N2), A/duck/HK/313/4/78 (H5N3), A/swine/Hokkaido/10/85(H3N2), A/WSN/33 (H1N1), and B/Lee/40. These studies bring the fact that strictinin acts directly with the viral particles and inhibit the early stage of viral entry and virus-induced hemifusion [87].

Haidari et al. studied pomegranate polyphenol extract (PPE) against H1N1 (A/USSR/90/77), H3N2 (A/Hong Kong/2/68, A/HK (H3N2)), and B (B/Harbin/07/94) viruses, which shows drastic proliferation inhibition without cytotoxicity in virus-adapted MDCK cells. H3N2-induced agglutination of chicken is inhibited by PPE suggesting the target was viral attachment. PPE showed direct inhibitory and virucidal effects on the H3N2 virus which are observed through the RT-PCR technique. The single-cycle and multiple-cycle growth conditions indicated PPE affects viral replication but did not alter vRNP entry and translocation in the host cell. To identify active potential components from the extract, four polyphenol compounds, ellagic acid, caffeic acid, luteolin, and punicalagin, were selected and examined. Surprisingly, the tannin compound punicalagin plays similar activities, like PPE, and mimics the same effect. In addition to that, PPE produces a synergic effect when combined with oseltamivir [126]..

The tannin-rich components tannic acid and pentagalloylglucose of *Hamamelis virginiana* of 1702 g/mol show inhibition of viral binding and neuraminidase [101].

#### Catechol

Hydroxytyrosol (HT) is one of the main phenolic components of *Olea eurolaea* belonging to the class of catechols produced a virucidal effect which was revealed in the study of Yamada et al. Hydroxytyrosol inactivates enveloped viruses including influenza A/Hokkaido/30/2000 (H1N1), A/Hokkaido/52/98 (H3N2), A/chicken/Yamaguchi/7/04 (H5N1), and A/chicken/Yokohama/aq55/01 (H9N2). HT did not show any cytotoxic effect on MDCK cells; it affected H9N2 virus NP protein synthesis and suppressed mRNA synthesis at 24 h.p.i. Electron microscopic analysis detects the structural disruption of the H9N2 virus by HT [113].

#### Anthraquinone

*Aloe vera* is a widely used herb of economical importance. In this study, two new anthraquinones, namely aloesaponarin-I (1) and aloesaponarin-II (2), were isolated from the roots. From aloesaponarin-I (1), six derivatives were obtained by the consecutive process like methylation (3), acetylation (4), and O-glycosyl (5, 6). As well as from aloesaponarin-II (2), a new derivative termed Tetra-O-acetyl-β-d-glucopyranosyl derivative obtained and these entire compounds experimented against H1N1 (A/Yucatán/2370/09, A/Mexico/InDRE797/10) infection. All these compounds (CC_50_ > 90 μM) did not exert any cytotoxic effect in MDCK cells. Virus replication inhibition analyzed by cytopathic effect reduction assay (CPE) noted compounds 3-(2′,3′,4′,6′-tetra-O-acetyl-β-d-glucopyranosyl-aloesaponarin-I (5) and 3-(2′,3′,4′,6′-tetra-O-acetyl-β-d-glucopyranosyl-aloesaponarin-II (7) against A/Yucatán/2370/09 (IC_50_ of 30.77 and 13.70 μM) and A/Mexico/InDRE797/10 (IC_50_ of 62.28 and 19.47 μM) reduction effect. Since other derivatives did not exhibit any antiviral effect, compounds 5 and 7 were taken for further studies. Plaque inhibition test using A/Yucatán/2370/09 strain noted the dose-dependent addition of compound 5 (50 and 100 μM) had significantly reduced the virus titer more than compound 7. The time-of-addition experiment appraising the efficacy of two compounds yielded a 70% reduction of virus titer at 6–10 h post-treatment [69].

#### Glycosides

Glycosides are active biological compounds reported in *Albizia julibrissin*, *Panax quinquefolium*, and *Panax ginseng* proved effective anti-influenza compounds.

Sun et al. isolated total saponin from the stem bark of *Albizia julibrissin* (AJSt) and fractionated it into AJS30, AJS50, AJS75, and AJS95. These compounds hemolytic activities and their adjuvant effect was reviewed. The compound AJSt, AJS50, AJS75, and AJS95 experienced minor hemolytic effects. The adjuvant effect of the compounds in the mice immunized with ovalbumin (OVA) and recombinant fowlpox virus vector–based avian influenza vaccine (rFPV) unveiled AJSt, AJS50, and AJS75 potent activities like enhancing concanavalin A (Con A), lipopolysaccharide (LPS), antigen-stimulated splenocyte proliferation, and serum antigen-specific IgG, IgG1, IgG2a, and IgG2b antibody titers. In-depth analysis of adjuvant action revealed AJS75 was the most potent adjuvant by inducing cellular and humoral response through stimulating cytokines and chemokines [68].

Dong et al. selected ginseng extracts (GE) and ginsenosides against the 2009 pandemic virus (A/Nanchang/8002/2009 H1N1 (NC2)) and observed the effect in both in vitro and in vivo study. The mice infected with GE-NC2 showed an increased survival rate and minimum weight loss. Similarly, the pretreated ginsenoside- and NC2 (Rb1-NC2)-received mice show a remarkable survival rate. Meanwhile, the mice at 3 d.p.i. show a reduction in viral titer which revealed ginsenoside action against lethal lung damage. Hemagglutination assay confirms the interaction of ginsenoside with the HA, thereby interfering with the virus attachment. The authors explore sugar moieties of ginseng play a crucial role in the interaction with HA of the virus. Oligosaccharide binding assay confirms that ginsenoside prevents virus attachment with α 2–3′ sialic acid receptors on the host cell surface. Subsequently, it minimizes virus entry and thereof decreases the infection [116].

Chan et al. chose special types of ginsenoside (Re, Rg1, and PPT) of *Panax ginseng* and studied the effect of inflammation and apoptosis induced by the H9N2 (A/Quail/Hong Kong/G1/97) virus. Human umbilical vein endothelial cells (HUVECs) were infected with H9N2 produce inflammation by inducing the chemokine IP-10 production. Ginsenoside PPT inhibits IP-10 production by regulating the micro RNA, miR-15b. Post-treatment of ginsenoside Re (50 μM) partially reduced the virus-adapted apoptosis which denotes its cytoprotection and increased cell viability effect [115].

#### Diarylheptanoids

Diarylheptanoids are structurally distinctive compounds with diverse biological and pharmacological effects [193]. Recently, its anti-influenza effects have been reported in *Alpinia officinarum* and *Curcuma longa*.

From the rhizome of *Alpinia officinarum*, ten diarylheptanoids were isolated and their anti-influenza efficiency against H1N1 (A/PR/8/34) examined which provide the EC_50_ value of diarylheptanoids was lower than CC_50_ and MNCC (maximum non-cytotoxic concentration). The diarylheptanoids 7-4″-hydroxy-3″-methoxyphenyl)-1-phenyl-4E-hepen-3-one (3) and (5S)-5-hydroxy-7-(4″-hydroxyphenyl)-1-phenyl-3-heptanone (8) show the EC_50_ values 2.9 ± 0.3 μg/mL and 0.7 ± 0.3 μg/mL were lower than the control ribavirin EC_50_ 16.7 ± 0.4 μg/mL. Hence, the authors suggested diarylheptanoids 3 and 8 as a potent source of anti-influenza activity [70].

The authors continued the study by orally administering these two compounds 7-4″-hydroxy-3″-methoxyphenyl)-1-phenyl-4E-hepen-3-one (AO-0002) and (5S)-5-hydroxy-7-(4″-hydroxyphenyl)-1-phenyl-3-heptanone (AO-0011) three times daily to the H1N1 (A/PR/8/34)-infected mice for 6 days after infection. AO-0002 (100 mg/kg) effectively reduced the bodyweight loss and enhanced the survival period of infected mice. AO-0002 (30 mg/kg, 30 and 100 mg/kg) notably reduced the virus titer in the lung BALF of infected mice on days 3 and 6 after infection. AO-0011 did not exhibit the above activities; hence, the author selected AO-0002 for further in vitro analysis. AO-0002 effectively inhibited the infection of H1N1 (A/PR/8/34, oseltamivir-resistant A/PR/8/34, A/Bangkok/93/03), H3N2 (A/Ishikawa/7/82, A/Fukushima/13/43), and B (B/Singapore/222/79, B/Fukushima/15/93) viruses in MDCK cells. Plaque reduction assay revealed AO-0002 was unable to interfere with the adsorption or invasion of the virus but dose-dependently (20–40 μg/mL) suppressed the expression of viral antigen and mRNA synthesis [71].

Chen et al. evaluated the efficiency of curcumin against H1N1 (A/Puerto Rico/8/34) and H6N1 (A/chicken/Taiwan/NCHU0507/99) which revealed no cytotoxic effect in virus-infected MDCK cells. Dose-dependent addition of curcumin reduced viral replication. Specifically, 30 μM of curcumin lessened 90% of virus yield. Time-of-addition experiments verified the direct inhibitory effect of curcumin. The mechanism was revealed by plaque reduction assay, which showed the early stage of inhibitory activity includes attachment prevention but not penetration. HA assay demonstrated curcumin possesses HA inhibition activity by interacting and interrupting the link between viruses HA and host cell receptors but not with the host RBC cell. In comparison with amantadine, curcumin did not evoke viral resistance [93].

*Curcuma longa* empowers a broad spectrum of antiviral activities. This creates interest for the investigators to investigate the presence of the substance in the herb. Recently, Dao et al. isolated 3 new (1–3) and 10 known (4–13) curcuminoids from the rhizome of *Curcuma longa* and performed neuraminidase inhibition assays against H1N1 (A/California/08/2009, A/Sw/Kor/CAH1/04, H274Y mutant) and H9N2 (A/Chicken/Korea/O1310/2001). From the study, all compounds are proven as non-competitive inhibitors with significant NA activity, of which compounds 4, 5, and 13 showed the best potent ability [94].

#### Isothiocyanate

Isothiocyanate is one of the active ingredients of *Wasabi japonica* which shows a significant virucidal effect against the influenza virus when treated with the composition of O.1SmL isothiocyanate in addition to 0.05 mL virus. The content of isothiocyanate differs in different parts of the plant where the rhizome (0.80 mol·mL^−1^) had a high amount compared with the fibrous root and petiole [148].

## Conclusion

Influenza remains a global threat. Even though it is under control, handling the pandemic situation remains a great challenge. Vaccine development and drug resistance coupled together to create a massive crisis in the difficult condition. To combat the problem, new drugs are necessary to practice. Keeping this view, alternative and traditional medicines provide new insights into the arrival of new drugs. This paper emphasizes an overview of the active ingredients of plants and their promising scientifically validated activity of the anti-influenza characteristics. Therefore, shortly, these active ingredients can be promoted for use as a new safe drug. A successful attempt can be claimed only when the clinical trial is appreciable.

## Data Availability

All information provided in the manuscript is obtained from the references.

## Abbreviations

ADV3: Adenovirus 3
AL-1: 14-a-Lipoyl andrographolide
AMPK: 5′ adenosine monophosphate-activated protein kinase
APS: *Astragalus* polysaccharide
AJSt: Total saponin from the stem bark of *Albizia julibrissin*
BALF: Bronchoalveolar lavage fluid
BCPS: *Bupleurum chinense* polysaccharides
BeA: Betulinic acid
CC_50_: Half maximal cytotoxic concentration
CDC: Centers for Disease Control and Prevention
CEF: Chick embryo fibroblasts
CLK1: cdc2-like kinase 1
Con A: Concanavalin A
CPE: Cytopathic effect reduction assay
CVB3: Coxsackie virus B3
DAP: 14-deoxy-11,12-dehydroandrographolide
DART TOF-MS: Direct analysis in real time mass spectrometry
diTPSs: Diterpene synthases
EC_50_: Half maximal effective concentration
EC: Epicatechin
ECG: Epicatechin-3-gallate
EGC: Epigallocatechin
EGCg: (−) Epigallocatechin gallate
EGCG: Epigallocatechin-3-gallate
EHext: Extract of *Ephedra herba*
ELS: Endosome and lysosome
ELISA: Enzyme-linked immunosorbent assay
ESI: Electrospray ionization
EtOAc: Ethyl acetate
EV71: Enterovirus 71
FDA: Food and Drug Administration
GE: Ginseng extract
GP: *Panax ginseng* polysaccharides
GTE: Green tea extract
HA: Hemagglutinin
7HC: 7-Hydroxycoumarin
HCP: *Houttuynia cordata* polysaccharides
HEF: Hemagglutinin esterase fusion
HeLa: Human epithelioid cervical carcinoma
HI: Hemagglutination inhibition assay
HIV: Human immunodeficiency virus
HPLC: High-performance liquid chromatography
HRV: Human rhinovirus
HT: Hydroxytyrosol
HUVECs: Human umbilical vein endothelial cells
IAV: Influenza A virus
IBV: Influenza B virus
IC: Inhibitory concentration
IC_50_: Half maximal inhibitory concentration
ICV: Influenza C virus
IDV: Influenza D virus
IFN: Interferon
IIV: Inactivated influenza vaccines
IRF-3: Interferon regulatory factor 3
IRPS: *Isatis indigotica* root polysaccharides
LAIV: Live attenuated influenza vaccines
LC: Liquid chromatography
LPS: Lipopolysaccharide
M1: Matrix protein
MDBK: Madin-Darby bovine kidney cells
MDCK: Madin-Darby bovine canine kidney cells
MeOH: Methanol
MNCC: Maximal non-cytotoxic concentration
MxA: Myxovirus resistance gene A
MS: Mass spectroscopy
MTT assay: 3-(4,5-Dimethylthiazol-2-yl)-2,5-diphenyltetrazolium bromide, a tetrazole) assay
NA: Neuraminidase
NEML: National essential medicines list
NF-κB: Nuclear factor-κB
NI: Neuraminidase inhibition assay
NIC: National Influenza Centers
NO: Nitric oxide
NP: Nucleoprotein or nucleocapsid protein
nsLTPs: Fetuin-binding non-specific lipid transfer protein
NTL: *Narcissus tazetta* lectin
OAS: 2-5′ oligoadenylate synthetase
OSMIP: Oseltamivir molecularly imprinted polymer
OVA: Ovalbumin
8-PK: 8-Prenylkaempferol
PA: Patchouli alcohol
PDB: Protein Data Bank
p.i.: Post-infection
PIV3: Parainfluenza virus 3
PPE: Pomegranate polyphenol extract
Q3R: Quercetin 3-rhamnoside
QIV: Quadrivalent inactivated vaccine
qRT-PCR: Real-time quantitative reverse transcription PCR
RANTES: Regulated upon Activation, Normal T cell Expressed and Secreted
rFPV: Recombinant fowlpox virus vector–based avian influenza vaccine
RSV: Respiratory syncytial virus
SAR: Structure-activity relationship
SIV: Swine influenza virus
SRB method: Sulforhodamine B assay (SRB)
T&CM: Traditional and Complementary medicine
TF3: Theaflavin digallate
TIV: Trivalent inactivated vaccine
TLC: Thin-layer chromatography
TOA: Time-of-addition assay
UF: Ultrafiltration
vRNP: Nuclear export of viral ribonucleoprotein
vRNP complex: Viral ribonucleoprotein complex
WHO: World Health Organization

## References

[1] Dacso CC, Couch RB, Six HR, Young JF, Quarles JM, Kasel JA (1984). Sporadic occurrence of zoonotic swine influenza virus infections. J Clin Microbiol.

[2] Simonsen L (1999). The global impact of influenza on morbidity and mortality. Vaccine.

[3] McCauley JW, Hongo S, Kaverin NV, Kochs G, Lamb RA, Matrosovich MN, Perez DR, Palese P, Presti RM, Rimstad E, King AMQ, Adams MJ, Carstens EB, Lefkowitz E (2012). Orthomyxoviridae. Virus taxonomy, classification and nomenclature of viruses: ninth report of the International Committee on Taxonomy of Viruses.

[4] Ortiz-Baez AS, Eden JS, Moritz C, Holmes EC (2020). A divergent Articulavirus in an Australian gecko identified using meta-transcriptomics and protein structure comparisons. Viruses.

[5] Bresee JS, Fry AM, Sambhara S, Cox NJ, Plotkin SA, Orenstein WA, Offit PA, Edwards KM (2018). Inactivated influenza vaccines. Plotkin’s Vaccines,7th edn.

[6] Wang B, Loeb M, Poland GA (2019). Influenza Vaccines—Are They Efficacious or Not?. Vaccinations.

[7] Long JS, Mistry B, Haslam SM, Barclay WS (2019). Host and viral determinants of influenza A virus species specificity.Nat. Rev Microbiol.

[8] Pellett PE, Mitra S, Holland TC, Tselis AC, Booss J (2014). Basics of virology. Handbook of clinical neurology.

[9] Zhai SL, Zhang H, Chen SN, Zhou X, Lin T, Liu R, Lv DH, Wen XH, Wei WK, Wang D, Li F (2017). Influenza D virus in animal species in Guangdong Province, southern China. Emerg Infect Dis.

[10] White SK, Ma W, McDaniel CJ, Gray GC, Lednicky JA (2016). Serologic evidence of exposure to influenza D virus among persons with occupational contact with cattle. J Clin Virol.

[11] Asha K, Kumar B (2019). Emerging influenza D virus threat: what we know so far. J Clin Med.

[12] Stohr K (2003). Overview of the WHO Global Influenza Programme. Dev Biol (Basel).

[13] Jester B, Schwerzmann J, Mustaquim D, Aden T, Brammer L, Humes R, Shult P, Shahangian S, Gubareva L, Xu X, Miller J (2018). Mapping of the US domestic influenza virologic surveillance landscape. Emerg Infect Dis.

[14] World Health Organization (2019) WHO Global Report on Traditional and Complementary Medicine. Available at: https://www.who.int/publications-detail/who-global-report-on-traditional-and-complementary-medicine-2019. Accessed May 3, 2020.

[15] Pan SY, Litscher G, Chan K, Yu ZL, Chen HQ, Ko KM (2014). Traditional medicines in the world: where to go next?. Evid Based Complement Alternat Med.

[16] Bouvier NM, Palese P (2008). The biology of influenza viruses. Vaccine.

[17] Yi Tsang N, Zhao LH, Wai Tsang S, Zhang HJ (2017). Antiviral Activity and Molecular Targets of Plant Natural Products Against Avian Influenza Virus. Curr Org Chem.

[18] Laver WG, Webster RG (1976). Preparation and immunogenicity of a purified influenza virus haemagglutinin and neuraminidase subunit vaccine. Postgrad Med J.

[19] Francis ME, King ML, Kelvin AA (2019). Back to the Future for Influenza Preimmunity-Looking Back at Influenza Virus History to Infer the Outcome of Future Infections. Viruses.

[20] Gao Q, Brydon EW, Palese P (2008). A seven-segmented influenza A virus expressing the influenza C virus glycoprotein HEF. J Virol.

[21] Stewart SM, Pekosz A (2012). The influenza C virus CM2 protein can alter intracellular pH, and its transmembrane domain can substitute for that of the influenza A virus M2 protein and support infectious virus production. J Virol.

[22] Matsuzaki Y, Sugawara K, Furuse Y, Shimotai Y, Hongo S, Oshitani H, Mizuta K, Nishimura H (2016). Genetic lineage and reassortment of influenza C viruses circulating between 1947 and 2014. J Virol.

[23] Nakatsu S, Murakami S, Shindo K, Horimoto T, Sagara H, Noda T, Kawaoka Y (2018). Influenza C and D viruses package eight organized ribonucleoprotein complexes. J Virol.

[24] Su S, Fu X, Li G, Kerlin F, Veit M (2017). Novel influenza D virus: epidemiology, pathology, evolution and biological characteristics. Virulence.

[25] Hilleman MR (1954). Antigenic variation of influenza viruses. Annu Rev Microbiol.

[26] Hill KL, Donelson JE, Brenner S, Miller JH (2001). Antigenic Variation. Encyclopedia of Genetics.

[27] Lamb A, Mahy BWJ, Van Regenmortel MHV (2008). Influenza. Encyclopedia of Virology, 3rd Edition.

[28] Ditmar MF, Polin RA, Ditmar MF (2011). Infectious Diseases. Pediatric Secrets, 5th Edition.

[29] Gerth HJ, Bauer KH, Steinitz H (1975). Is there evidence for antigenic drift of influenza C virus?. Zentralbl Bakteriol Parasitenkd Infekt Hyg.

[30] Treanor J (2004). Influenza vaccine—outmaneuvering antigenic shift and drift. N Engl J Med.

[31] Centers for Disease Control and Prevention (2018) Immunization: The Basics definition of terms. Available at: https://www.cdc.gov/vaccines/vac-gen/imz-basics.htm. Accessed May 16, 2018

[32] Ulmer J, Valley U, Rappuoli R (2006). Vaccine manufacturing: challenges and solutions. Nat Biotechnol.

[33] Palese P (2006). Making better influenza virus vaccines?. Emerg Infect Dis.

[34] Krammer F, Palese P (2015). Advances in the development of influenza virus vaccines. Nat Rev Drug Discov.

[35] Moa AM, Muscatello DJ, Turner RM, MacIntyre CR (2017). Epidemiology of influenza B in Australia: 2001-2014 influenza seasons. Influenza Other Respir Viruses.

[36] Halasa NB, Gerber MA, Berry AA, Anderson EL, Winokur P, Keyserling H, Eckard AR, Hill H, Wolff MC, McNeal MM, Edwards KM, Bernstein DI (2015). Safety and Immunogenicity of Full-Dose Trivalent Inactivated Influenza Vaccine (TIV) Compared With Half-Dose TIV Administered to Children 6 Through 35 Months of Age. J Pediatric Infect Dis Soc.

[37] Zhang K, Wu X, Shi Y, Gou X, Huang J (2021). Immunogenicity of H5N1 influenza vaccines in elderly adults: a systematic review and meta-analysis. Hum Vaccin Immunother.

[38] Centers for Disease Control and Prevention (2018). Seasonal Flu Shot. Available at: https://www.cdc.gov/flu/prevent/flushot.htm. Accessed May 16, 2018.

[39] Wong SS, Webby RJ (2013). Traditional and new influenza vaccines. Clin Microbiol Rev.

[40] Robertson CA, DiazGranados CA, Decker MD, Chit A, Mercer M, Greenberg DP (2016). Fluzone® High-Dose Influenza Vaccine. Expert Rev Vaccines.

[41] Sullivan SJ, Jacobson R, Poland GA (2010). Advances in the vaccination of the elderly against influenza: role of a high-dose vaccine. Expert Rev Vaccines.

[42] Overton ET (2012). Sometimes, more is better. J. Infect. Dis..

[43] Tregoning JS, Russell RF, Kinnear E (2018). Adjuvanted influenza vaccines. Hum Vaccin Immunother.

[44] Centers for Disease Control and Prevention (2021). Live Attenuated Influenza Vaccine [LAIV] (The Nasal Spray Flu Vaccine) Available at:https://www.cdc.gov/flu/prevent/nasalspray.htm. Accessed January 25, 2021

[45] Clancy S (2008). Genetics of the influenza virus. Nature Education.

[46] Madsen A, Cox RJ (2020). Prospects and challenges in the development of universal influenza vaccines. Vaccines.

[47] Pica N, Palese P (2013). Toward a universal influenza virus vaccine: prospects and challenges. Annu Rev Med.

[48] Wei CJ, Crank MC, Shiver J, Graham BS, Mascola JR, Nabel GJ (2020). Next-generation influenza vaccines: opportunities and challenges. Nat Rev Drug Discov.

[49] Houser K, Subbarao K (2015). Influenza vaccines: challenges and solutions. Cell Host Microbe..

[50] Science.gov (2021) Oak Ridge, TN, US. https://www.science.gov/topicpages/v/vaccine+manufacturing+capacity

[51] McLean KA, Goldin S, Nannei C, Sparrow E, Torelli G (2016). The 2015 global production capacity of seasonal and pandemic influenza vaccine. Vaccine.

[52] Plans P (2008). Recommendations for the prevention and treatment of influenza using antiviral drugs based on cost-effectiveness. Expert Rev Pharmacoecon Outcomes Res.

[53] Shie JJ, Fang JM (2019). Development of effective anti-influenza drugs: congeners and conjugates - a review. J Biomed Sci.

[54] Influenza (flu) antiviral drugs and related information (2020) US Food and Drug Administration, Rockville. https://www.fda.gov/drugs/information-drug-class/influenza-flu-antiviral-drugs-and-related-information#ApprovedDrugs. Accessed October 26,2020

[55] Kohno S, Kida H, Mizuguchi M, Hirotsu N, Ishida T, Kadota J, Shimada J (2011). Intravenous peramivir for treatment of influenza A and B virus infection in high-risk patients. Antimicrob Agents Chemother.

[56] Allen UD, Aoki FY, Stiver HG (2006). The use of antiviral drugs for influenza: recommended guidelines for practitioners. Can J Infect Dis Med Microbiol.

[57] Hayden FG, Sugaya N, Hirotsu N, Lee N, de Jong MD, Hurt AC, Ishida T, Sekino H, Yamada K, Portsmouth S, Kawaguchi K, Shishido T, Arai M, Tsuchiya K, Uehara T, Watanabe A, Baloxavir Marboxil Investigators G (2018). Baloxavir marboxil for uncomplicated influenza in adults and adolescents. N Engl J Med.

[58] Mishin VP, Patel MC, Chesnokov A, De La Cruz J, Nguyen HT, Lollis L, Hodges E, Jang Y, Barnes J, Uyeki T, Davis CT, Wentworth DE, Gubareva LV (2019). Susceptibility of influenza A, B, C, and D viruses to baloxavir (1). Emerg Infect Dis.

[59] Amarelle L, Lecuona E, Sznajder JI (2017). Anti-influenza treatment: drugs currently used and under development. Arch Bronconeumol.

[60] De Clercq E (2006). Antiviral agents active against influenza A viruses. Nat Rev Drug Discov..

[61] World Health Organization (2010) Pandemic (H1N1) 2009: antiviral drug resistance. Available at: https://www.who.int/csr/disease/swineflu/frequently_asked_questions/ antivirals/resistance/en/. Accessed 29 Dec 2010.

[62] Norberg P, Lindh M, Olofsson S (2015). Published sequences do not support transfer of oseltamivir resistance mutations from avian to human influenza A virus strains. BMC Infect Dis.

[63] Pal SK, Shukla Y (2003). Herbal medicine: current status and the future. Asian Pac J Cancer Prev..

[64] Ganjhu RK, Mudgal PP, Maity H, Dowarha D, Devadiga S, Nag S, Arunkumar G (2015). Herbal plants and plant preparations as remedial approach for viral diseases. VirusDis.

[65] Mousa HA (2017). Prevention and treatment of influenza, influenza-like illness, and common cold by herbal, complementary, and natural therapies. J Evid Based Complementary Altern Med..

[66] Kamboj VP (2000). Herbal medicine. Curr Sci.

[67] Arora R, Chawla R, Marwah R, Arora P, Sharma R, Kaushik V, Goel R, Kaur A, Silambarasan M, Tripathi R (2011). Potential of complementary and alternative medicine in preventive management of novel H1N1 flu (Swine flu) pandemic: thwarting potential disasters in the bud. Evid Based Complement Altern Med.

[68] Sun H, He S, Shi M (2014). Adjuvant-active fraction from *Albizia julibrissin* saponins improves immune responses by inducing cytokine and chemokine at the site of injection. Int Immunopharmacol.

[69] Borges-Argáez R, Chan-Balan R, Cetina-Montejo L, Ayora-Talavera G, Sansores-Peraza P, Gómez-Carballo J, Cáceres-Farfán M (2019). In vitro evaluation of anthraquinones from *Aloe vera* (*Aloe barbadensis* Miller) roots and several derivatives against strains of influenza virus. Ind Crops Prod.

[70] Sawamura R, Sun Y, Yasukawa K, Shimizu T, Watanabe W, Kurokawa M (2010). Antiviral activities of diarylheptanoids against influenza virus in vitro. J Nat Med.

[71] Sawamura R, Shimizu T, Sun Y, Yasukawa K, Miura M, Toriyama M, Motohashi S, Watanabe W, Konno K, Kurokawa M (2010). *In vitro* and *in vivo* anti-influenza virus activity of diarylheptanoids isolated from *Alpinia officinarum*. Antivir Chem Chemother.

[72] Chen JX, Xue HJ, Ye WC, Fang BH, Liu YH, Yuan SH, Yu P, Wang YQ (2009). Activity of andrographolide and its derivatives against influenza virus in vivo and in vitro. Biol Pharm Bull.

[73] Cai W, Li Y, Chen S, Wang M, Zhang A, Zhou H, Chen H, Jin M (2015). 14-Deoxy-11, 12-dehydroandrographolide exerts anti-influenza A virus activity and inhibits replication of H5N1 virus by restraining nuclear export of viral ribonucleoprotein complexes. Antiviral Res.

[74] Hayashi K, Narutaki K, Nagaoka Y, Hayashi T, Uesato S (2010). Therapeutic effect of arctiin and arctigenin in immunocompetent and immunocompromised mice infected with influenza A virus. Biolo Pharm Bull.

[75] Park S, Kim JI, Lee I, Lee S, Hwang MW, Bae JY, Heo J, Kim D, Han SZ, Park MS (2013). *Aronia melanocarpa* and its components demonstrate antiviral activity against influenza viruses. Biochem Biophys Res Commun.

[76] Kallon S, Li X, Ji J, Chen C, Xi Q, Chang S, Xue C, Ma J, Xie Q, Zhang Y (2013). *Astragalus* polysaccharide enhances immunity and inhibits H9N2 avian influenza virus in vitro and in vivo. J Animal Sci Biotechnol.

[77] Ahmad A, Javed MR, Rao AQ, Husnain T (2016). Designing and screening of universal drug from neem (*Azadirachta indica*) and standard drug chemicals against influenza virus nucleoprotein. BMC complement Altern Med.

[78] Zhang XQ, Chen HS (1989) Immuno-pharmacological effects of Bupleurum chinense polysaccharide. Chinese J Pharmacol Toxicol 3(1):30–33

[79] Liu AL, Shu SH, Qin HL, Lee SM, Wang YT, Du GH (2009). In vitro anti-influenza viral activities of constituents from *Caesalpinia sappan*. Planta Med.

[80] Jeong HJ, Kim YM, Kim JH, Kim JY, Park JY, Park SJ, Ryu YB, Lee WS (2012). Homoisoflavonoids from *Caesalpinia sappan* displaying viral neuraminidases inhibition. Biol Pharm Bull.

[81] Matsumoto K, Yamada H, Takuma N, Niino H, Sagesaka YM (2011). Effects of green tea catechins and theanine on preventing influenza infection among healthcare workers: a randomized controlled trial. BMC Complement Altern Med.

[82] Imanishi N, Tuji Y, Katada Y, Maruhashi M, Konosu S, Mantani N, Terasawa K, Ochiai H (2002). Additional inhibitory effect of tea extract on the growth of influenza A and B viruses in MDCK cells. Microbiol Immunol.

[83] Song JM, Lee KH, Seong BL (2005). Antiviral effect of catechins in green tea on influenza virus. Antiviral Res.

[84] Kaihatsu K, Mori S, Matsumura H, Daidoji T, Kawakami C, Kurata H, Nakaya T, Kato N (2009). Broad and potent anti-influenza virus spectrum of epigallocatechin-3-O-gallate-monopalmitate. J Mol Genet Med.

[85] Nakayama M, Suzuki K, Toda M, Okubo S, Hara Y, Shimamura T (1993). Inhibition of the infectivity of influenza virus by tea polyphenols. Antiviral Res.

[86] Sahoo M, Jena L, Rath SN, Kumar S (2016). Identification of suitable natural inhibitor against influenza A (H1N1) neuraminidase protein by molecular docking. Genomics Inform.

[87] Saha RK, Takahashi T, Kurebayashi Y, Fukushima K, Minami A, Kinbara N, Ichitani M, Sagesaka YM, Suzuki T (2010). Antiviral effect of strictinin on influenza virus replication. Antiviral Res.

[88] Zhang L, Cheng YX, Liu AL, Wang HD, Wang YL, Du GH (2010). Antioxidant, anti-inflammatory and anti-influenza properties of components from *Chaenomeles speciosa*. Molecules.

[89] Hayashi K, Imanishi N, Kashiwayama Y, Kawano A, Terasawa K, Shimada Y, Ochiai H (2007). Inhibitory effect of cinnamaldehyde, derived from Cinnamomi cortex, on the growth of influenza A/PR/8 virus in vitro and in vivo. Antiviral Res.

[90] Kurokawa M, Kumeda CA, Yamamura JI, Kamiyama T, Shiraki K (1998). Antipyretic activity of cinnamyl derivatives and related compounds in influenza virus-infected mice. Eur J Pharmacol.

[91] Kurokawa M, Watanabe W, Shimizu T, Sawamura R, Shiraki K (2010). Modulation of cytokine production by 7-hydroxycoumarin in vitro and its efficacy against influenza infection in mice. Antiviral Res.

[92] Liao Q, Qian Z, Liu R, An L, Chen X (2013). Germacrone inhibits early stages of influenza virus infection. Antiviral Res.

[93] Chen DY, Shien JH, Tiley L, Chiou SS, Wang SY, Chang TJ, Lee YJ, Chan KW, Hsu WL (2010). Curcumin inhibits influenza virus infection and haemagglutination activity. Food Chem.

[94] Dao TT, Nguyen PH, Won HK, Kim EH, Park J, Won BY, Oh WK (2012). Curcuminoids from Curcuma longa and their inhibitory activities on influenza A neuraminidases. Food Chem.

[95] Li R, Liu T, Liu M, Chen F, Liu S, Yang J (2017). Anti-influenza A virus activity of dendrobine and its mechanism of action. J Agric Food Chem.

[96] Liu AL, Liu B, Qin HL, Lee SM, Wang YT, Du GH (2008). Anti-influenza virus activities of flavonoids from the medicinal plant *Elsholtzia rugulosa*. Planta Med.

[97] Mantani N, Imanishi N, Kawamata H, Terasawa K, Ochiai H (2001). Inhibitory effect of (+)-catechin on the growth of influenza A/PR/8 virus in MDCK cells. Planta Med.

[98] Miki K, Nagai T, Suzuki K, Tsujimura R, Koyama K, Kinoshita K, Furuhata K, Yamada H, Takahashi K (2007). Anti-influenza virus activity of biflavonoids. Bioorg Med Chem Lett.

[99] Dao TT, Nguyen PH, Lee HS, Kim E, Park J, Lim SI, Oh WK (2011). Chalcones as novel influenza A (H1N1) neuraminidase inhibitors from *Glycyrrhiza inflata*. Bioorg Med Chem Lett.

[100] Ryu YB, Kim JH, Park SJ, Chang JS, Rho MC, Bae KH, Park KH, Lee WS (2010). Inhibition of neuraminidase activity by polyphenol compounds isolated from the roots of *Glycyrrhiza uralensis*. Bioorg Med Chem Lett.

[101] Theisen LL, Erdelmeier CA, Spoden GA, Boukhallouk F, Sausy A, Florin L, Muller CP (2014). Tannins from *Hamamelis virginiana* bark extract: characterization and improvement of the antiviral efficacy against influenza A virus and human papillomavirus. PLoS one.

[102] Hayashi K, Kamiya M, Hayashi T (1995). Virucidal effects of the steam distillate from *Houttuynia cordata* and its components on HSV-1, influenza virus and HIV. Planta Med.

[103] Zhu H, Lu X, Ling L, Li H, Ou Y, Shi X, Lu Y, Zhang Y, Chen D (2018). *Houttuynia cordata* polysaccharides ameliorate pneumonia severity and intestinal injury in mice with influenza virus infection. J Ethnopharmacol.

[104] Choi HJ, Song JH, Park KS, Kwon DH (2009). Inhibitory effects of quercetin 3-rhamnoside on influenza A virus replication. Eur J Pharm Sci.

[105] Wang X, Xue Y, Li Y, Liu F, Yan Y, Zhang H, Jin Q (2018). Effects of *Isatis* root polysaccharide in mice infected with H3N2 swine influenza virus. Res Vet Sci.

[106] Mak NK, Leung CY, Wei XY, Shen XL, Wong RN, Leung KN, Fung MC (2004). Inhibition of RANTES expression by indirubin in influenza virus-infected human bronchial epithelial cells. Biochem Pharmacol.

[107] Yang Z, Wang Y, Zheng Z, Zhao S, Zhao JI, Lin Q, Li C, Zhu Q, Zhong N (2013). Antiviral activity of Isatis indigotica root-derived clemastanin B against human and avian influenza A and B viruses in vitro. Int J Mol Med.

[108] He J, Qi WB, Wang L, Tian J, Jiao PR, Liu GQ, Ye WC, Liao M (2013). Amaryllidaceae alkaloids inhibit nuclear-to-cytoplasmic export of ribonucleoprotein (RNP) complex of highly pathogenic avian influenza virus H5N1. Influenza Other Respi Viruses.

[109] He J, Qi W, Tian J, Jiao P, Liu G, Zhang C, Liao M (2012). Amaryllidaceae alkaloids exhibit anti-influenza activity in MDCK cells, an investigation of Amaryllidaceae Alkaloids and MDCK cells insight. J Anim Vet Adv.

[110] Ooi VEC, Chan PKS, Chiu LCM, Sun SSM, Wong HNC (2014). Antiviral activity of Chinese medicine - derived phytochemicals against avian influenza A ( H5N1 ) virus. Hong Kong Med J.

[111] Ooi LS, Ho WS, Ngai KL, Tian L, Chan PK, Sun SS, Ooi VE (2010). *Narcissus tazetta* lectin shows strong inhibitory effects against respiratory syncytial virus, influenza A (H1N1, H3N2, H5N1) and B viruses. J Biosci.

[112] Ooi LSM, Tian L, Su M, Ho WS, Sun SSM, Chung HY, Wong HNC, Ooi VEC (2008). Isolation, characterization, molecular cloning and modeling of a new lipid transfer protein with antiviral and antiproliferative activities from *Narcissus tazetta*. Peptides.

[113] Yamada K, Ogawa H, Hara A, Yoshida Y, Yonezawa Y, Karibe K, Nghia VB, Yoshimura H, Yamamoto Y, Yamada M, Nakamura K, Imai K (2009). Mechanism of the antiviral effect of hydroxytyrosol on influenza virus appears to involve morphological change of the virus. Antiviral Res.

[114] Yoo DG, Kim MC, Park MK, Park KM, Quan FS, Song JM, Wee JJ, Wang BZ, Cho YK, Compans RW, Kang SM (2012). Protective effect of ginseng polysaccharides on influenza viral infection. PLoS One.

[115] Chan LY, Kwok HH, Chan RW, Peiris MJ, Mak NK, Wong RN, Chan MC, Yue PY (2011). Dual functions of ginsenosides in protecting human endothelial cells against influenza H9N2-induced inflammation and apoptosis. J Ethnopharmacol.

[116] Dong W, Farooqui A, Leon AJ, Kelvin DJ (2017). Inhibition of influenza A virus infection by ginsenosides. PLoS One.

[117] Ooi LS, Sun SS, Ooi VE (2004). Purification and characterization of a new antiviral protein from the leaves of *Pandanus amaryllifolius* (Pandanaceae). Int J Biochem Cell Biol.

[118] Ha TJ, Lee MH, Park CH, Kim JI, Oh E, Pae SB, Park JE, Kim SU, Kwak DY (2018). rvH1N1 neuraminidase inhibitory activities of phenolics from *Perilla frutescens* (L.) and their contents in cultivars and germplasm. Plant Breed Biotech.

[119] Kang J, Liu C, Wang H, Li B, Li C, Chen R, Liu A (2014). Studies on the bioactive flavonoids isolated from *Pithecellobium clypearia* Benth. Molecules.

[120] Li C, Xu LJ, Lian WW, Pang XC, Jia H, Liu AL, Du GH (2018). Anti-influenza effect and action mechanisms of the chemical constituent gallocatechin-7-gallate from *Pithecellobium clypearia* Benth. Acta Pharmacol Sin.

[121] Yu Y, Zhang Y, Wang S, Liu W, Hao C, Wang W (2019). Inhibition effects of patchouli alcohol against influenza a virus through targeting cellular PI3K/Akt and ERK/MAPK signaling pathways. Virol J.

[122] Li YC, Peng SZ, Chen HM, Zhang FX, Xu PP, Xie JH, He JJ, Chen JN, Lai XP, Su ZR (2012). Oral administration of patchouli alcohol isolated from Pogostemonis Herba augments protection against influenza viral infection in mice. Int Immunopharmacol.

[123] Wu H, Li B, Wang X, Jin M, Wang G (2011). Inhibitory effect and possible mechanism of action of patchouli alcohol against influenza A (H2N2) virus. Molecules.

[124] Chen KT, Zhou WL, Liu JW, Zu M, He ZN, Du GH, Chen WW, Liu AL (2012). Active neuraminidase constituents of Polygonum cuspidatum against influenza A(H1N1) influenza virus. Zhongguo Zhong Yao Za Zhi.

[125] Shoji M, Arakaki Y, Esumi T, Kohnomi S, Yamamoto C, Suzuki Y, Takahashi E, Konishi S, Kido H, Kuzuhara T (2015). Bakuchiol Is a Phenolic Isoprenoid with Novel Enantiomer-selective Anti-influenza A Virus Activity Involving Nrf2 Activation. J Biol Chem.

[126] Haidari M, Ali M, Casscells SW, Madjid M (2009). Pomegranate (*Punica granatum*) purified polyphenol extract inhibits influenza virus and has a synergistic effect with oseltamivir. Phytomedine.

[127] Jeong HJ, Ryu YB, Park SJ, Kim JH, Kwon HJ, Kim JH, Park KH, Rho MC, Lee WS (2009). Neuraminidase inhibitory activities of flavonols isolated from *Rhodiola rosea* roots and their in vitro anti-influenza viral activities. Bioorg Med Chem.

[128] Knox YM, Suzutani T, Yosida I, Azuma M (2003). Anti-influenza virus activity of crude extract of *Ribes nigrum* L. Phytother Res.

[129] Knox YM, Hayashi K, Suzutani T, Ogasawara M, Yoshida I, Shiina R, Tsukui A, Terahara N, Azuma M (2001). Activity of anthocyanins from fruit extract of Ribes nigrum L. against influenza A and B viruses. Acta Virol.

[130] Roschek B, Fink RC, McMichael MD, Li D, Alberte RS (2009). Elderberry flavonoids bind to and prevent H1N1 infection in vitro. Phytochemistry.

[131] Li Y, Jiang R, Ooi LS, But PP, Ooi VE (2007). Antiviral triterpenoids from the medicinal plant *Schefflera heptaphylla*. Phytother Res.

[132] Ding Y, Dou J, Teng Z, Yu J, Wang T, Lu N, Wang H, Zhou C (2014). Antiviral activity of baicalin against influenza A (H1N1/H3N2) virus in cell culture and in mice and its inhibition of neuraminidase. Arch Virol.

[133] Nagai T, Miyaichi Y, Tomimori T, Suzuki Y, Yamada H (1990). Inhibition of influenza virus sialidase and anti-influenza virus activity by plant flavonoids. Chem Pharm Bull.

[134] Nagai T, Miyaichi Y, Tomimori T, Suzuki Y, Yamada H (1992). In vivo anti-influenza virus activity of plant flavonoids possessing inhibitory activity for influenza virus sialidase. Antiviral Res.

[135] Nagai T, Suzuki Y, Tomimori T, Yamada H (1995). Antiviral activity of plant flavonoid, 5,7,4′-trihydroxy-8-methoxyflavone, from the roots of *Scutellaria baicalensis* against influenza A (H3N2) and B viruses. Biol Pharm Bull.

[136] Nagai T, Moriguchi R, Suzuki Y, Tomimori T, Yamada H (1995). Mode of action of the anti-influenza virus activity of plant flavonoid, 5, 7, 4′-trihydroxy-8-methoxyflavone, from the roots of *Scutellaria baicalensis*. Antiviral Res.

[137] Seong RK, Kim JA, Shin OS (2018). Wogonin, a flavonoid isolated from *Scutellaria baicalensis*, has anti-viral activities against influenza infection via modulation of AMPK pathways. Acta Virol.

[138] Hour MJ, Huang SH, Chang CY, Lin YK, Wang CY, Chang YS (2013) Lin CW (2013) Baicalein, ethyl acetate, and chloroform extracts of *Scutellaria baicalensis* inhibit the neuraminidase activity of pandemic 2009 H1N1 and seasonal influenza A viruses. Evid Based Complement Alternat Med. 10.1155/2013/750803 10.1155/2013/750803 PMC3705751 23864896

[139] Hayashi K, Mori M, Matsutani Knox Y, Suzutan T, Ogasawara M, Yoshida I, Hosokawa K, Tsukui A, Azum M (2003). Anti influenza virus activity of a red-fleshed potato anthocyanin. Food Sci Technol Res.

[140] Dang Z, Jung K, Zhu L, Lai W, Xie H, Lee KH, Huang L, Chen CH (2014). Identification and synthesis of quinolizidines with anti-influenza A virus activity. ACS Med Chem Lett.

[141] Yang YJ, Li JY, Liu XW, Zhang JY, Liu YR, Li B (2013). A non-biological method for screening active components against influenza virus from traditional Chinese medicine by coupling a LC column with oseltamivir molecularly imprinted polymers. PLoS One.

[142] Ryu YB, Curtis-Long MJ, Kim JH, Jeong SH, Yang MS, Lee KW, Lee WS, Park KH (2008). Pterocarpans and flavanones from *Sophora flavescens* displaying potent neuraminidase inhibition. Bioorg Med Chem Lett.

[143] Chiou WF, Chen CC, Wei BL (2011). 8-Prenylkaempferol suppresses influenza A virus-induced RANTES production in A549 cells via blocking PI3K-mediated transcriptional activation of NF-κB and IRF3. Evid Based Complement Alternat Med.

[144] Ma JY, Zhao DR, Yang T, Liu D, Li RT, Li HM (2019). Prenylflavanones isolated from *Sophora flavescens*. Phytochem Lett.

[145] Hsieh CF, Chen YL, Lin CF, Ho JY, Huang CH, Chiu CH, Hsieh PW, Horng JT (2016). An extract from *Taxodium distichum* targets hemagglutinin- and neuraminidase-related activities of influenza virus in vitro. Sci Rep.

[146] Ibrahim AK, Youssef AI, Arafa AS, Foad R, Radwan MM, Ross S, Hassanean HA, Ahmed SA (2013). Anti-H5N1 virus new diglyceride ester from the Red Sea grass *Thallasodendron ciliatum*. Nat Prod Res.

[147] Mohammed MM, Hamdy AH, El-Fiky NM, Mettwally WS, El-Beih AA, Kobayashi N (2014). Anti-influenza A virus activity of a new dihydrochalcone diglycoside isolated from the Egyptian seagrass *Thalassodendron ciliatum* (Forsk.) den Hartog. Nat Prod Res.

[148] Hu SQ, Hu G (2011) Extraction of isothiocyanate in Wasabi Japonica Matsum and its effects to influenza virus. In: 2011 IEEE International Symposium on IT in Medicine and Education, December 2011. Vol 1, Guangzhou, China, pp 45–48. 10.1109/ITiME.2011.6130780

[149] Cai Z, Zhang G, Tang B, Liu Y, Fu X, Zhang X (2015). Promising Anti-influenza Properties of Active Constituent of *Withania somnifera* Ayurvedic Herb in Targeting Neuraminidase of H1N1 Influenza: Computational Study. Cell Biochem Biophysics.

[150] Hong EH, Song JH, Kang KB, Sung SH, Ko HJ, Yang H (2015). Anti-influenza activity of betulinic acid from *Zizyphus jujuba* on influenza A/PR/8 virus. Biomol Ther.

[151] Gangopadhyay AD, Ganguli SA, Datta AB (2011). Inhibiting H5N1 hemagglutinin with samll molecule ligands. Int J Bioinformatics Res.

[152] Tiwari S (2008). Plants: A rich source of herbal medicine. J Nat Prod.

[153] Trouvelot S, Héloir MC, Poinssot B, Gauthier A, Paris F, Guillier C, Combier M, Trdá L, Daire X, Adrian M (2014). Carbohydrates in plant immunity and plant protection: roles and potential application as foliar sprays. Front Plant Sci.

[154] Berg JM, Tymoczko JL, Stryer L (2002) In: Freeman WH (ed) Monosaccharides are aldehydes or ketones with multiple hydroxyl groups. Biochemistry, 5th edn, New York Available from: https://www.ncbi.nlm.nih.gov/books/NBK22547/. Accessed 20 Sep 2020

[155] Shariatinia Z, Hasnain MS, Nayak AK (2019). Pharmaceutical applications of natural polysaccharides. Natural Polysaccharides in Drug Delivery and Biomedical Applications..

[156] Redasani VK, Bari SB, Redasani VK, Bari SB (2015). Approaches for Prodrugs. Prodrug Design.

[157] Habtemariam S, Habtemariam S (2019). Medicinal Foods As Potential Therapies for Type-2 Diabetes and Associated Diseases. Introduction to plant secondary metabolites—From biosynthesis to chemistry and antidiabetic action.

[158] Ghosh S, Chisti Y, Banerjee UC (2012). Production of shikimic acid. Biotechnol Adv.

[159] Bochkove DV, Sysolyatin SV, Kalashnikov AI, Surmachena IA (2011). Shikimic acid: review of its analytical, isolation and purification techniques from plant and microbial sources. J Chem Biol.

[160] Franzoni G, Trivellini A, Bulgari R, Cocetta G, Ferrante A, Khan MIR, Reddy PS, Ferrante A, Khan NA (2019). Bioactive Molecules as Regulatory Signals in Plant Responses to Abiotic Stresses. Plant Signaling Molecules.

[161] Pott DM, Osorio S, Vallarino JG (2019). From central to specialized metabolism: an overview of some secondary compounds derived from the primary metabolism for their role in conferring nutritional and organoleptic characteristics to fruit. Front Plant Sci.

[162] Ncube B, Van Staden J (2015). Tilting Plant Metabolism for Improved Metabolite Biosynthesis and Enhanced Human Benefit. Molecules.

[163] Shoker RM (2020). A Review Article: The Importance of the Major groups of Plants Secondary Metabolism Phenols, Alkaloids, and Terpenes. Int J Appl Sci Biotechnol.

[164] Singh B, Sharma RA (2015). Plant terpenes: defense responses, phylogenetic analysis, regulation and clinical applications. 3. Biotech.

[165] Perveen S, Perveen S, Al-Taweel A (2018). Introductory Chapter: Terpenes and Terpenoids. Terpenes and Terpenoids.

[166] Connolly JD, Hill RA (1991). Dictionary of terpenoids.

[167] Wang G, Tang W, Bidigare RR, Demain AL (2005). Terpenoids as therapeutic drugs and pharmaceutical agents. Zhang L.

[168] Nguyen TD, MacNevin G, Ro DK, Hopwood DA (2012). De novo synthesis of high-value plant sesquiterpenoids in yeast. Methods in enzymology, vol 517.

[169] Jones B (2017). Diterpenoids: Types, Functions and Research.

[170] Zerbe P, Chiang A, Dullat H, O’Neil-Johnson M, Starks C, Hamberger B, Bohlmann J (2014). Diterpene synthases of the biosynthetic system of medicinally active diterpenoids in *Marrubium vulgare*. Plant J.

[171] Garg A, Sharma R, Dey P, Kundu A, Kim HS, Bhakta T, Kumar A, Silva AS, Nabavi SF, Saeedi M, Nabavi SM (2020). Analysis of triterpenes and triterpenoids. Recent Advances in Natural Products Analysis.

[172] Richard T, Temsamani H, Cantos-Villar E, Monti JP, Rolin D (2013). Chapter Two - Application of LC–MS and LC–NMR Techniques for Secondary Metabolite Identification. Advances in botanical research, vol 67.

[173] Lattanzio V, Ramawat KG, Merillon JM (2013). Phenolic compounds: introduction. Nat Prod.

[174] Kumar S, Sumner B, Sumner LW, Liu HW, Begley TP (2020). Modern plant metabolomics for the discovery and characterization of natural products and their biosynthetic genes. Compr Nat Prod III.

[175] Cheynier V (2012). Phenolic compounds: from plants to foods. Phytochem Rev.

[176] Rauter AP, Ennis M, Hellwich KH, Herold BJ, Horton D, Moss GP, Schomburg I (2018). Nomenclature of flavonoids (IUPAC recommendations 2017). Pure Appl Chem.

[177] Jan S, Abbas N, Jan S, Abbas N (2018). Chapter 4—Chemistry of himalayan phytochemicals. Himalayan Phytochemicals Amsterdam.

[178] Kim HP, Park H, Son KH, Chang HW, Kang SS (2008). Biochemical pharmacology of biflavonoids: implications for anti-inflammatory action. Arch Pharm Res.

[179] Chen X, Mukwaya E, Wong MS, Zhang Y (2014). A systematic review on biological activities of prenylated flavonoids. Pharm Biol.

[180] Amawi H, Ashby CR, Tiwari AK (2017). Cancer chemoprevention through dietary flavonoids: what’s limiting?. Chin J Cancer.

[181] Zakaryan H, Arabyan E, Oo A, Zandi K (2017). Flavonoids: promising natural compounds against viral infections. Arch Virol.

[182] Panche AN, Diwan AD, Chandra SR (2016). Flavonoids: an overview. J Nutr Sci.

[183] Glanz VY, Myasoedova VA, Grechko AV, Orekhov AN (2018). Inhibition of sialidase activity as a therapeutic approach. Drug Des Devel Ther.

[184] Yiannakopoulou EC (2012). Recent patents on antibacterial, antifungal and antiviral properties of tea. Recent Pat Antiinfect Drug Discov.

[185] Chacko SM, Thambi PT, Kuttan R, Nishigaki I (2010). Beneficial effects of green tea: a literature review. Chin Med.

[186] Kosugi Y (2020) Green tea consumption In: World Green Tea Association Available at: https://www.o-cha.net/english/teacha/distribution/greentea3.html. Accessed 15 Aug 2020.

[187] Park M, Yamada H, Matsushita K, Kaji S, Goto T, Okada Y, Kosuge K, Kitagawa T (2011). Green tea consumption is inversely associated with the incidence of influenza infection among schoolchildren in a tea plantation area of Japan. J Nutrit.

[188] Malla A, Ramalingam S, Grumezescu AM, Holban AM (2018). Chapter 11—Health Perspectives of an Isoflavonoid Genistein and its Quantification in Economically Important Plants. Role of Materials Science in Food Bioengineering.

[189] Foudah AI, Abdel-Kader MS, Justino J (2017). Isoflavonoids. Flavonoids - From Biosynthesis to Human Health.

[190] Sharma V, Ramawat KG, Ramawat K, Mérillon JM (2013). Isoflavonoids. Natural Products.

[191] Mottaghipisheh J, Stuppner H (2021). A Comprehensive Review on Chemotaxonomic and Phytochemical Aspects of Homoisoflavonoids, as Rare Flavonoid Derivatives. Int J Mol Sci.

[192] Vicente AR, Manganaris GA, Sozzi GO, Crisosto CH, Florkowski WJ, Prussia SE, Shewfelt SL, Brueckner B (2009). Nutritional quality of fruits and vegetables. Postharvest handling: a systems approach, Food Science and Technology Series.

[193] Sun DJ, Zhu LJ, Zhao YQ, Zhen YQ, Zhang L, Lin CC, Chen LX (2020). Diarylheptanoid: A privileged structure in drug discovery. Fitoterapia.

